# Multilevel Racism and Discrimination and Cardiovascular Disease and Related Biopsychosocial Mechanisms: An Integrated Scoping and Literature Review and Future Research Agenda

**DOI:** 10.1007/s11886-025-02238-3

**Published:** 2025-06-04

**Authors:** Danielle L. Beatty Moody, Elizabeth J. Pantesco, Ayla Novruz, Nedelina Tchangalova, Richard C. Sadler, Kellee White Whilby, Jason Ashe, Gilbert C. Gee, LaBarron K. Hill, Shari R. Waldstein

**Affiliations:** 1 School of Social Work, Rutgers University, New Brunswick, NJ USA; 2 (Department of Psychological and Brain Sciences), Villanova University, Villanova, PA USA; 3 (Department of Psychology), University of Maryland, Baltimore County, Baltimore, MD USA; 4 STEM Library, University of Maryland Libraries, College Park, MD USA; 5 Departments of Public Health and Family Medicine, Michigan State University, Flint, MI USA; 6 School of Public Health, Department of Health Policy and Management, University of Maryland, College Park, MD USA; 7 School of Public Health, Department of Community Health Sciences, University of California, Los Angeles, CA United States; 8 (Department of Psychology), North Carolina A & T University, Greensboro, NC USA

**Keywords:** Multilevel racism, Discrimination, Cardiovascular racial disparities, Subclinical and clinical cardiovascular disease, African Americans

## Abstract

**Purpose of Review:**

In the last two decades, empirical research has significantly advanced our understanding of the link between discrimination and cardiovascular disease (CVD). This integrated scoping and narrative literature review delineates the extant peer-reviewed research on discrimination and clinical and subclinical CVD in samples that include Black adults, using a multilevel conceptualization of race-related discrimination and racism. We also identify potential intermediary mechanisms in the racism-CVD relationship and propose a comprehensive future research agenda.

**Recent Findings:**

Using the Population, Exposure and Outcome framework and PRISMA guidelines, we identified 37 empirical reports for inclusion drawn from 1900 to 2024. The bulk of the literature has focused on discrimination and racism that occurs at the interpersonal level (28 studies), while a smaller but growing body of work has examined cultural (5 studies) or institutional and structural-level racism and discrimination (4 studies) in relation to CVD risk. The majority of these studies show that greater exposure to discrimination or racism is associated with increased clinical or subclinical CVD risk. Potential pathways include societal, environmental, psychological, and biological factors; however, few studies have conducted formal tests of mediation.

**Summary:**

The literature suggests robust relations of multilevel racism and discrimination to manifestations of CVD across diverse exposure and outcome measures in Black adults. Our recommendations to eliminate cardiovascular health inequities in Black communities include enhancing academic scholarship training, securing targeted and protected funding, and adopting more robust methodological approaches.

**Supplementary Information:**

The online version contains supplementary material available at 10.1007/s11886-025-02238-3.

## Introduction

In the United States (U.S.), striking racial disparities exist in cardiovascular disease (CVD), including coronary heart disease (CHD), cerebrovascular disease (CeD), peripheral arterial disease (PAD), and sudden cardiac death [1]. Specifically, African Americans^1^ or Black Americans of African descent living in the U.S. represent 13.4% of the population yet have the highest CVD prevalence and incidence of all racial/ethnic groups [2]. African Americans are twice as likely to experience sudden cardiac death, and they are less likely to survive to hospital discharge than Whites (25.2% vs. 37.4%) [3]. African Americans have almost twice the risk of an initial stroke, and by 2032, are expected to experience a 134% increase in stroke compared with a 91% increase in Whites [4–6]. This increased CVD burden contributes to African Americans’ shorter life expectancy compared to Whites. For instance, the rates of premature CHD and stroke mortality (i.e., death among persons aged < 75 years) per 100,000 were 65.5 and 25.0, respectively, in African Americans compared with 43.2 and 10.02 in White Americans [7]. Notably, the onset of these stark disparities is much earlier in adulthood for African Americans compared to Whites as they are experiencing at least twice the risk of CVD-related mortality in young adulthood [8].

Disparities in traditional cardiovascular risk factors (e.g., hypertension, diabetes mellitus, obesity) are significant drivers of the earlier onset and elevated CVD rates among African Americans. Yet, reports from the American Heart Association (AHA), Centers for Disease Control and Prevention (CDC), National Academies of Sciences, Engineering, and Medicine, National Institutes of Health (NIH), Healthy People 2030, and Circulation [9–15], all clearly document that the most significant opportunities for reducing disproportionate burden, disability, and death from CVD in the U.S. lie not solely in our ability to identify and modify biomedical risk factors, but in our efforts to mitigate the *social* determinants of CVD that unevenly impact minoritized racial/ethnic groups, particularly African Americans [16]. Indeed, these calls are further underscored by a 2024 AHA policy statement [17]—Addressing Structural Racism Through Public Policy Advocacy—that serves as a “forward-looking blueprint” to drive a broad spectrum of stakeholders seeking to address structural racism to achieve health equity in the context of cardiovascular health.

In this regard, race- and ethnicity-related health inequities—such as those seen in CVD—reveal entrenched, multilevel, race-based discrimination. The term *race* as a construct does not reflect a biological or genetic basis for health differences, but instead functions wholly as a historically architected construct that continues to imbue social, economic, and political hierarchies and, in turn, marginalization in American society [18]. Specifically, the racial groups we recognize today were socially developed in tandem with and in service of European colonial expansion in the Americas, with the largest initial influx of African Americans brought to the U.S. involuntarily during the transatlantic slave trade [19]. Thus, race is a manifestation of racism, which from its inception has shaped the lived experience and well-being of African Americans in the U.S. [20].

The last three decades have seen explosive growth in research seeking to elucidate the relations of racism and discrimination to CVD as demonstrated by several reviews and meta-analyses [21–26]. Yet, because the predominant emphasis in this work has been on blood pressure (BP) and hypertension, there remains a need to examine the body of work explicating the linkages of racism and discrimination to additional clinical and subclinical CVD endpoints. In this review, we first define and examine the constructs of racism and discrimination. Then, we review the empirical reports exploring the relations of racism and discrimination to clinical (i.e., stroke, transient ischemic attack, coronary artery disease (CAD), CHD, PAD, sudden cardiac arrest and CVD-related mortality) and subclinical (i.e., atherosclerosis, endothelial dysfunction, subclinical CeD, and coronary artery calcification) CVD. While often used interchangeably, CAD and CHD are not synonymous. CAD refers specifically to the buildup of plaque inside the coronary arteries, typically diagnosed through imaging methods like angiography [27]. In contrast, CHD is a broader term that includes CAD but also includes angina, myocardial infarction, and other ischemic heart conditions. This work is theoretically centered on the understanding that racism and race-based discrimination can serve as direct forms of psychosocial stress with physiological consequences [28, 29]. Thus, we next consider mechanistic pathways in the relations of racism and discrimination to CVD. We also discuss limitations of the current evidence and share guidance for future research. In this review, we advocate for the need to concurrently examine racism and discrimination as *multidimensional* and *multilevel* constructs which act as fundamental determinants of CVD pathogenesis. Finally, we outline critical next steps for how CVD health disparities research may be enhanced, along with opportunities for healthcare to mitigate these linkages.

## Discrimination and Racism: Defining Two Critical Social Determinants of Health

Racism—the mechanism that enshrines white supremacy—operates at multiple levels, ranging from individual, cultural, and institutional to structural (see Table 1; [some authors have posited even more levels; see [28, 30–32]). Here, we provide descriptions of each level.

**Table 1:** Summary of Levels of Racism, Definitions, Dimensions, and Examples

Level of Racism			
Structural	The totality of how society is organized in a manner that privileges whites at the expense of non-White, racialized groups Structural racism is widely understood to be the root origin and driving force behind race as a social hierarchy and in turn *all* levels of racism [26, 30, 33, 34] Reflected via the interconnections among macrolevel systems, ideologies, institutions, social forces and other processes that create, sustain, and advance race-based inequities across society for non-White racialized groups [35]		
	**Definitions**	**Dimensions**	**Examples**
Institutional	Practices and policies of organizations that create and perpetuate racial inequity and the race-based hierarchy [35]	- Built and social environment (e.g., residential, housing, and neighborhood contexts) - Justice system (e.g., law enforcement, courts, and corrections) - Labor sector (e.g., job market, employment, and workplace environment) - Education system (e.g., K-12 and postsecondary) - Immigration system (e.g., policies, practices) - Political system (e.g., local, state, and federal government practices, policies, and participation) [36] - Healthcare system (e.g., insurance companies, hospital systems, and independent providers) - Mass media system (e.g., television broadcast and film, video, music, radio, cinema, newspaper and journalism, magazines, and internet)	- Discrimination by lending institutions against minority homebuyers - Violence by police against innocent people of color - School policies that disenfranchise students of color
Cultural	Tangible and non-tangible aspects of society, such as language, symbols, media, and assumptions, grounded in historical and/or contemporary white supremacy [37–40]	- Pervasively negative and unfair depictions, of stereotypes, prejudicial attitudes and beliefs, and tropes in society via media outlets and other institutions - Area-level prejudice [40] - Public symbols and representations signifying honor or support for events, practices, or figures with known pro-racist histories or agendas, or denigrative misrepresentations or tropes of cultural or racial/ethnic groups racism [41]	- Statues honoring figures with an established pro-racist history (e.g., statues of Nathan Bedford Forrest, Samuel Sullivan Cox, or the Pioneers Monument) - Sports teams names and mascots (e.g., Redskins, Chiefs, Savages, Hurons, Redmen) - Online hate speech [42] - Implicit racial bias
Interpersonal	Individual experiences of unfair treatment related to one’s (perceived) racial group membership, in the context of everyday social interactions)	- Direct, implicit, or vicarious unfair experiences of occurring across various domains of society (e.g., workplace, health care, school, from law enforcement, or in public settings) - Comprises a continuum of events including expectations of future experiences, microaggressions, slights, indignities to property, threat, and physical and bodily harm [43]	- Not being hired or promoted for a job - Being discouraged by a teacher or advisor from continuing your education - Being treated with less respect

The predominant focus of empirical research has centered on self-reported experiences of racism and discrimination that have either unfolded in interpersonal interactions or from the perspective of the individual. At this level, various forms of terminology have been used including, interpersonal racism and racial discrimination. Use of the term *interpersonal racism* often centers on prejudicial attitudes and discriminatory behaviors ranging from microaggressions to hate crimes, whereas the term *racial discrimination*, is more reflective of the unfair, differential, and sometimes even violent treatment of a person based on their perceived race, ethnicity, ancestry or national origin. Decades of research have documented a marked burden of interpersonal racism among African Americans which is higher than most other racial/ethnic groups in the U.S., with upwards of 80% of African Americans reporting some experience of racial discrimination in their lifetime [44–48]. African Americans describe these experiences as burdensome [49–51] and as occurring across major life domains (e.g., in the judicial system, from law enforcement, when seeking healthcare [52–56]), as well as in everyday activities (e.g., while shopping, at work, or at school). Importantly, day-to-day interpersonal discrimination not explicitly linked to race/ethnicity, often referred to as *everyday discrimination*, is also higher among African Americans (e.g., see [47, 57]). In addition, the prevalence and types of discrimination that African Americans experience is shaped by other key demographics, including, age, sex, gender, and socioeconomic status (SES) [58]. Indeed, the differential patterning of discrimination in relation to other social identities may create even greater disproportionate risk among individuals with multiple marginalized identities, and thusly, is important to understand in the context of CVD risk (e.g., see [59, 60]). For example, elucidating differential experiences with discrimination among subgroups of African Americans—such as lower SES African American women and mid-level SES African American men—can provide greater insight into the burden of discrimination, highlight which subgroups are more likely to experience particular types of discrimination, and reveal more specific linkages to health outcomes (e.g., see [61, 62]).

**Fig. 1:**
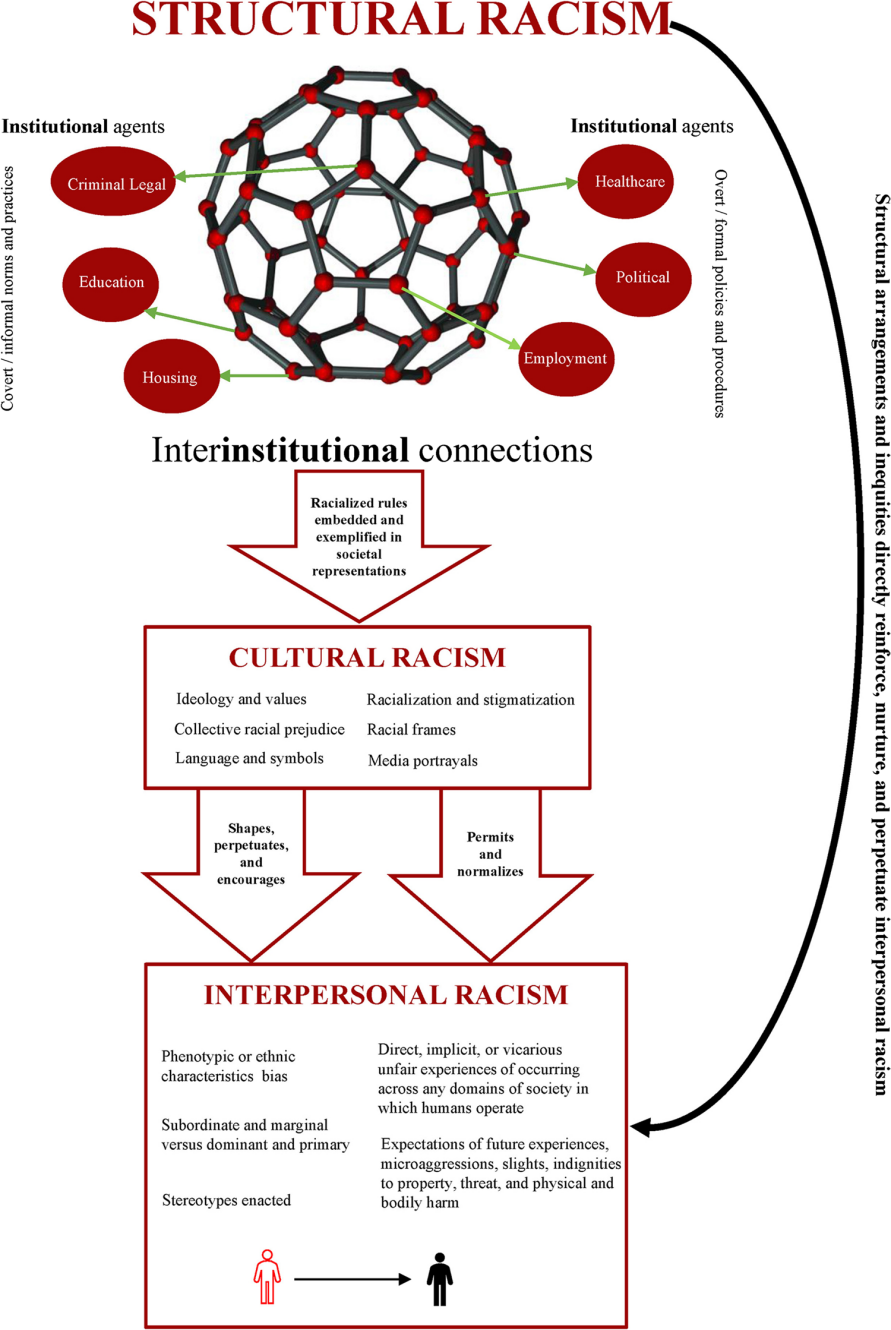
Relationships among structural, institutional, cultural, and interpersonal racism and the related flow of power to depict the “inter-institutional” connections among these levels of racism. (Adapted from Micheals et al. [71], with permission from Wiley Global Permissions)

In contrast to interpersonal-level racism and discrimination, work elucidating the contributions of cultural- and institutional-levels of racism is just beginning to emerge in the context of CVD health disparities. *Cultural racism* embodies the established ideologies of superiority in the values, language, imagery, symbols, and implicit assumptions of the larger society and its institutions [37] and can be reflected in the racist names of sports teams and mascots (e.g., Redskins) and hate speech on online social platforms such as X (formerly Twitter) and Facebook [63–65]. *Institutional racism* refers to the racial biases and discriminatory actions promulgated by social organizations, such as banks, courts, and governmental entities. The key insight here is that racial bias is not merely enacted by a few individuals, but also by powerful institutions that can shape access to social resources. These entities can limit one’s educational attainment, determine where one lives, and restrict access to rights such as the ability to vote. Whereas institutional racism refers to individual organizations, *structural racism* can be conceptualized as the interconnections across multiple organizations that work in concert to maintain white supremacy [35]. This occurs when one institution protects, provides resources, or assists another. For example, the police force may be discriminatory against Black persons, representing institutional racism (for recent example see, [66, 67]). However, courts and policy makers may afford protections to the police, thus constituting structural racism. As illustrated in Fig. 1, *structural racism* is widely understood to be the root force that sustains and perpetuates race as a social hierarchy and in turn shapes *all* other levels of racism as its sole purpose is to maintain the historically-founded power structure and race-based hierarchies [32–34, 68]; for further detailed review, see [26, 28, 69, 70].

## Research Evidence Linking Multilevel Racism and Discrimination to Clinical and Subclinical CVD

In this study, we used a mixed-methods approach drawing from two types of reviews: a scoping review and a narrative literature review. Initially, we followed the broad and systematic steps of a scoping review [72] to gather and review the wide range of studies on our topic.

Our search strategy was informed by the Population, Exposure, Outcome (PEO) framework [73]. The Public Health Librarian (NT) in consultation with the primary author (DLBM) identified key terms for three main concepts: *African Americans and/or Blacks* (P), *discrimination and/or racism* (E), and *cardiovascular disease* (O). Synonyms and related terms for each concept are presented in Appendix A, along with the search strategy in Appendix B. We searched four databases through EBSCO platform (Academic Search Ultimate, APA PsycINFO, CINAL, MEDLINE) and one through ProQuest (Public Health). Our aim was to search all relevant databases thoroughly, adhering to the scoping review guidelines to ensure comprehensive coverage of the subject.

**Fig. 2:**
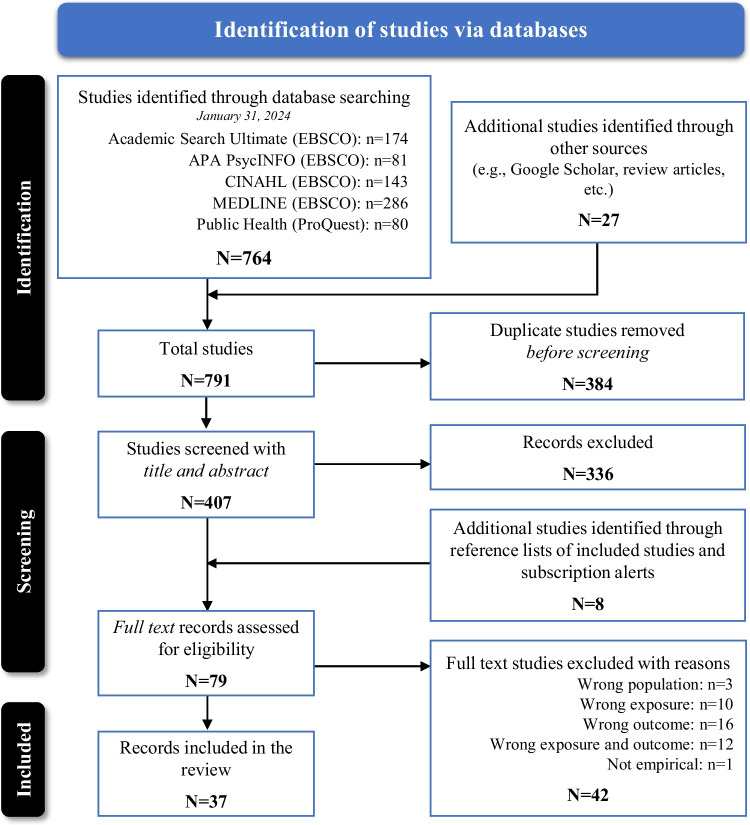
PRISMA showing flow of identification of final 37 studies included in this review

To meet eligibility, we reviewed all original English language peer-reviewed studies conducted in the U.S. that examined the relationship of discrimination and/or racism to clinical or subclinical CVD in samples that included African Americans. Studies related to children and adolescents (birth to 18 years) were excluded as the origins of those conditions are typically congenital in nature [74] and this review is focused on the roles of multilevel racism and discrimination in increased adulthood CVD outcomes. Given the extensive literature examining the relation of segregation to health, including empirical studies and reviews linking it to subclinical and clinical CVD endpoints and risk factors (e.g., [75–81]), this marker of structural racism was also excluded. Similarly, given the extensive examination of racism and discrimination in relation to blood pressure, hypertension, or other traditional cardiovascular risk factors (e.g., obesity, diabetes/glucose, cholesterol), these endpoints were also excluded (e.g., see [23, 25, 82–85]). Our selection process entailed the exportation of all unique records to the literature review software Rayyan for title/abstract screening [86]. All titles and abstracts were initially screened for inclusion by a primary reviewer (DLBM or EJP). In instances where inclusions criteria were unclear, the primary reviewer consulted with a second reviewer to ensure consistency and adherence to the inclusion criteria. Any remaining discrepancies were resolved through discussion with a third reviewer until a final decision was achieved. Full text of selected studies was obtained by the librarian (NT) and reviewed by authors (DLBM; EJP). The selection process is illustrated in Fig. 2.

After collecting the initial data from 37 studies included in this review (Appendix C), we then applied more detailed analysis methods typical for literature reviews to critically examine and synthesize existing literature, including critical evaluation, thematic analysis, synthesis of findings, and identification of gaps. This approach helped us to explore in-depth specific topics that emerged during the initial phase. By merging these two methods, our study was both wide-ranging and detailed, allowing us to compile a more complete analysis of the data collected.

Table 2 provides an overview of studies that examine indicators of discrimination and racism in relation to clinical and subclinical CVD outcomes in U.S.-based African American adult samples. We organize our findings by conceptual levels of discrimination and racism: structural and institutional, cultural, and interpersonal.

**Table 2:** Summary of select research linking multilevel and multidimensional discrimination and racism with subclinical and clinical cardiovascular disease (CVD) outcomes in African Americans

CVD-Related Outcome(s)	Reference	Study Population, Years, and Site(s)	Study Design	Measure(s) of Discrimination	Covariates	Relevant Tested Mediator(s)	Relevant Tested Moderator(s)	Findings
A) Individual-Level Discrimination and Racism and Measured Clinical CVD								
CVD mortality (atherosclerotic coronary heart disease, stroke, other atherosclerotic disease, or other cardiovascular disease)	Lawrence et al. (2023) [87]	Black (*n* = 1,633), White (*n* = 2,473), and Hispanic/Latino (*n* = 1,403) participants from the Multi-Ethnic Study of Atherosclerosis (MESA), 2002–2018. Multi-state sample.* N* = 5,509, mean age (overall, any discrimination) = 60.1	Longitudinal	-Everyday discrimination -Lifetime discrimination -Racial and ethnic residential segregation	Age, gender*, race/ethnicity, highest educational attainment, family income, nativity status, health insurance coverage, marital status, physical activity, smoking, alcohol use, BMI, hypertension status, diabetes status, duration lived in the neighborhood, neighborhood social cohesion, and neighborhood poverty	-Behavioral (e.g., smoking status and physical activity) -Clinical (e.g., BMI, hypertension status, and fasting glucose/diabetes)	-Gender* -Race/ethnicity -Racial and ethnic residential segregation	-**Full sample analyses:** Experiencing lifetime discrimination was associated with increased cardiovascular mortality for the overall population. Experiencing everyday discrimination was also associated with higher risks of cardiovascular mortality -**Race-stratified analyses:** Lifetime discrimination was associated with higher cardiovascular mortality in all racial groups. Black participants experienced the strongest associations between discrimination and increased cardiovascular mortality. However, everyday discrimination was not associated with increased cardiovascular mortality in Black participants, but an association was observed among White and Hispanic/Latino participants, although these CIs were wide and overlapped the null
CVD-specific mortality	Albert et al. (2010) [88]	Black women from the Black Women’s Health Study (BWHS), 1997–2005. Multi-state sample*. N* = 48,924, mean age = 40.5	Longitudinal	-Everyday discrimination -Institutionalized racism exposure	Age, education, occupation, health insurance status, marital status, physical activity, smoking status, alcohol use, BMI, HTN, diabetes, hyperlipidemia history, MI family history, neighborhood racial composition, and urbanization status	–	–	**Full sample analyses:** No associations found
Incident CVD (CHD, stroke, HF, and/or CVD death)	Dunlay et al. (2017) [89]	African American participants from the Jackson Heart Study, 2000–2012. Jackson, MS. *N* = 5,085, age range = 21–94	Longitudinal	-Everyday discrimination -Lifetime discrimination -Lifetime discrimination burden	Age, sex*, education, income, occupation, smoking status, BMI, HTN, diabetes, hypercholesterolemia, EGFR, perceived standing in community, healthcare assess, and social support (marital status, number of friends/relatives, and involvement in community groups)	–	-Age -Sex*	**Full sample analyses:** For African Americans, there was no significant association of discrimination (all measures) with incident CHD, incident stroke, or HF hospitalization. No differences were observed in these associations by age or sex
Incident CVD (MI, resuscitated cardiac arrest, coronary revascularization, definite angina, fatal or nonfatal stroke, and/or CVD death)	Everson-Rose et al. (2015) [90]	Black (non-Hispanic) (*n* = 1,718), White (non-Hispanic) (*n* = 2,538), Chinese (*n* = 794), and Hispanic/Latino (*n* = 1,451) participants from the Multi-Ethnic Study of Atherosclerosis (MESA), 2000–2011. Multi-state sample. *N* = 6,508, age range = 45–84	Longitudinal	-Everyday discrimination -Lifetime discrimination exposure with follow-up assessment of discrimination attribution (e.g., race)	Age, sex*, race, education, income, marital status, physical activity, smoking status, alcohol use, BMI, HTN or lipid-lowering medication, diabetes, SBP, lipid and triglyceride levels, chronic stress, and depressive symptoms (CES-D), and MESA field center	–	-Age -Sex* -Race	-**Full sample analyses:** In models with continuous discrimination variables, greater discrimination (for both measures: everyday and lifetime) was associated with greater CVD incidence, after adjusting for all covariates except ones related to stress and depressive symptoms. After adjusting for stress and depressive symptoms, greater discrimination (both measures) no longer had a significant association with CVD incidence. In models with categorical discrimination variables, greater lifetime discrimination (but not everyday discrimination) was associated with greater CVD incidence -**Sex interactions:** Greater everyday discrimination was associated with greater CVD incidence in men, but not women -No interactions observed between lifetime discrimination and race, age, or sex or between everyday discrimination and race and age
Incident stroke (incident and definite cases)	Sheehy et al. (2023) [91]	Black women from the Black Women’s Health Study (BWHS), 1997–2019. Multi-state sample. *N* = 48,375, median age = 38, age range = 21–69	Longitudinal	Perceived interpersonal racism	Age, education, insurance status, neighborhood SES, vigorous physical activity, cigarette smoking, BMI, history of hypertension, history of diabetes, history of CHD, history of hyperlipidemia, history of depression, and indicators for healthcare utilization	–	-Age -State of residence (e.g., Alabama, Arkansas, Georgia, Louisiana, Mississippi, North Carolina, South Carolina, and Tennessee) -Education -Neighborhood SES	-For Black women, experiencing interpersonal racism in situations involving employment, housing, and interactions with police was associated with increased incident stroke cases, after adjusting for demographic and vascular risk factors -Elevated stroke risk was also present in analyses restricted to definite stroke cases
-Severe coronary obstruction risk (via nuclear imaging study) -Moderate/severe (vs mild/none) coronary obstruction (via coronary angiogram)	Ayotte et al. (2012) [27]	Black (*n* = 164) and White (*n* = 629) male veteran participants from the Cardiac Decision Making Study, 1999–2001. Multi-site sample.* N* = 793, mean age = 63.2 (Black) and 60.6 (White)	Cross-sectional	Racial discrimination	Age, education, current smoking status, HTN, diabetes, prior revascularization or MI, negative affect, optimism, social support, and religiosity	–	–	**Race-stratified analyses only:** For Black participants, greater discrimination was associated with greater severe obstruction risk (similar results in patients who underwent coronary angiography) when controlling for clinical and psychosocial variables. No associations were found for White participants
B) Individual-Level Discrimination and Racism and Measured Subclinical CVD								
Arterial stiffness (via pulse wave velocity)	Bromfield et al. (2020) [92]	Black (*n* = 205) and White/Other (*n* = 108) post-myocardial infarction (MI) patients from the Myocardial Infarction and Mental Stress 2 study (MIMS2), 2011–2020. Emory-affiliated hospitals, Atlanta, GA.* N* = 313, age ≤ 60 years, mean age = 51.6	Cross-sectional	Everyday discrimination	Age, gender*, race, education, income, marital status, physical activity, BMI, CHD severity score, depressive symptoms (BDI-II), and perceived stress (PSS)	–	-Gender* -Race	**-Full sample analyses:** No associations found -**Race- and gender-stratified analyses**: For Black women, greater discrimination was associated with greater arterial stiffness. No associations found in other subgroups -**Race, gender, and everyday discrimination three-way interaction:** No effects observed
CAC (presence of coronary artery calcium)	Everage et al. (2012) [93]	American Black men (*n* = 571) and women (*n* = 791) from the Coronary Artery Risk Development in Young Adults (CARDIA) study, 1985–2000. Multi-state sample. *N* = 1,362, mean age = 39.7	Cross-sectional	Racial discrimination	Age, gender*, SES (education and income), BMI, HTN medication, diabetes, SBP, total cholesterol, trait anger, reactive responding, and depressive symptoms (CES-D)	–	Gender*	**Full sample analyses:** For Black Americans, greater discrimination was associated with lower odds of CAC presence. No significant gender interactions were observed
CAC	Cardarelli et al. (2010) [94]	African American (non-Hispanic) (*n* = 167), White (non-Hispanic) (*n* = 142), Hispanic (*n* = 183), and Other (*n* = 8) participants from the North Texas Heart (NTHH) Study, 2006–2008. Dallas/Fort Worth metropolitan areas, TX. *N* = 510, age = 45 years and older	Cross-sectional	-Racial discrimination -Response to unfair treatment	Age, gender*, race/ethnicity, education, smoking status, BMI, HTN, diabetes, hyperlipidemia, and history of CHD in first degree relative	–	Race/ethnicity	**Full sample analyses:** No significant main effect was observed for discrimination on CAC. Among those reporting discrimination, a passive response to unfair treatment was associated with the presence of CAC. No significant race interactions were found
-CAC -Coronary and abdominal aortic atherosclerosis (aortic plaque area and wall thickness) -Prevalent CAC (score ≥ 10 Agatston units) -CRP	Albert et al. (2008) [95]	African American (*n* = 790), White (*n* = 494), and Hispanic (*n* = 191) participants from the Dallas Heart Study (DHS), 1997–2005. Dallas, TX. *N* = 1,475, age range = 30–65	Cross-sectional	Racial/ethnic discrimination exposure	Age, gender*, education, physical activity, smoking status, BMI, HTN, diabetes, LDL-C, HDL-C, family history of MI, statin and aspirin use	–	–	**-Full sample analyses:** No associations observed between perceived discrimination and CAC or CRP. -**Gender- and education-stratified analyses:** No associations observed between perceived discrimination and CAC or CRP
CAC	Lewis et al. (2006) [96]	African American women from the Study of Women’s Health Across the Nation (SWAN), 1996–2003. Multi-state sample. *N* = 181, age range = 42–52 at baseline	Longitudinal	-Chronic everyday discrimination (averaged across 5 years) -Recent everyday discrimination (assessed at time of EBT scan) -Attribution (race/ethnicity vs. other) for everyday discrimination	Education, BMI, Framingham Risk Score (FRS; composite of age, SBP, total cholesterol, HDL-c, and current smoking status), and study site	–	–	**Full sample analyses:** For African American women, greater chronic everyday discrimination was associated with presence of CAC. No associations were found for recent everyday discrimination or attributions
CMIT (carotid intimal-medial thickness)	Cundiff et al. (2023) [97]	Black (*n* = 83) and White (*n* = 400) healthy working adults sampled from the Adult Health and Behavior Project—Phase 2 (AHAB-II), 2008–2011. Pittsburgh, PA. *N* = 483, age range = 30–54, mean age = 42.8	Cross-sectional	-Expectations of respect and appreciation (mean score) -General positivity and negativity of social interactions (mean score) -Neuroticism (NEO-PI-R) -Trait optimism (LOT-R) -Exposure to discrimination (PEDQ)	Age, sex, education (highest level completed), income, primary job title and responsibilities, smoking status, mean systolic blood pressure, standard lipid panel, and fasting serum glucose	–	Race	-Reporting greater expectations of respect and appreciation from others was associated with less preclinical vascular disease (CIMT). -These associations held true regardless of age, sex, race, SES, general negativity and positivity of daily social interactions, neuroticism, optimism, exposure to discrimination, mean systolic blood pressure, standard lipid panel, fasting serum glucose, and smoking status
CIMT	Beatty Moody et al. (2020) [98]	African American (*n* = 1,097) and White (*n* = 844) participants from the Healthy Aging in Neighborhoods of Diversity Across the Life Span (HANDLS) SCAN Study (MRI Ancillary Project of HANDLS), 2004–2009. Baltimore, MA. *N* = 1,941, age range = 30–64	Cross-sectional	-Everyday discrimination -Frequency of discrimination across sources	Age, sex*, SES, cigarette use, alcohol and drug use, BMI, HTN, diabetes, total cholesterol, CVD, lipid-lowering medication, and depressive symptoms (CES-D)	–	Depressive symptoms (CES-D)	**Race-stratified analyses:** For African American participants, greater discrimination (both measures) were associated with greater CIMT, which was more pronounced in those with greater depressive symptoms. No associations were found in White participants
CIMT	Lewis et al. (2019) [99]	Non-Hispanic African American women free of self-reported CVD and CVD equivalents. 2009–2010. Yale University Hospital Research Unit, New Haven, CT. *N* = 52, age range = 35–50	Cross-sectional	Expectations of future experiences with racism	Age, education, BMI, SBP, DBP, racial discrimination, depressive symptoms (BDI), chronic stress burden, and hostility	–	Past racial discrimination	**Full sample analyses:** For African American women, the expectation of future experiences with racism was associated with greater levels of CIMT, independent of past racial discrimination. No significant interaction of expectations of future experiences and past racial discrimination was observed
-CIMT -Left Ventricular Hypertrophy (LVH)	Okhomina et al. (2018) [100]	African American participants from the Jackson Heart Study, 2000–2004. Jackson, MS. *N* = 3,029, age range = 21–84	Cross-sectional	-Everyday discrimination (modified) -Lifetime discrimination -Lifetime discrimination burden (subsample reporting > 1 lifetime discrimination experience)	Age, education, income, occupation, smoking history, alcohol use, BMI, and HTN	–	Sex*	**Full sample analyses**: In the main effects models, no significant associations were found between everyday and lifetime discrimination and median CIMT and LVH. After full adjustment, experiencing high (vs. no) lifetime discrimination burden was associated with lower odds of LVH.—In the fully adjusted model, reporting active (vs passive) coping with everyday discrimination was associated with a greater odds of LVH for African American women. However, sex did not modify the associations of coping with lifetime discrimination with CIMT or LVH
-CIMT -Adventitial diameter	Peterson et al. (2016) [101]	African American (*n* = 283), White (*n* = 580), Chinese (*n* = 142), and Hispanic (*n* = 51) women from the Study of Women’s Health Across the Nation (SWAN), 1996–2007. Multi-state sample.* N* = 1,056, age range = 42–52	Longitudinal	Everyday discrimination (cumulative unfair treatment)	Age, race, household income, SES, physical activity, cigarette and alcohol use, BMI, DBP, insulin activity, cholesterol, triglycerides, and anticoagulant medication	–	Race	**Race interaction:** In race-stratified models, White women had greater discrimination/unfair treatment associated with greater CIMT and adventitial diameter. No association was found in African American, Chinese, or Hispanic participants
CIMT	Troxel et al. (2003) [102]	African American (*n* = 109) and White (*n* = 225) women from the Study of Women’s Health Across the Nation (SWAN), 1994–1997. Pittsburgh, PA site only.* N* = 334, age range = 42–52	Cross-sectional	-Everyday discrimination -Attribution to race/ethnicity for everyday discrimination	Age, BMI, and HDL-c	-BMI -HDL-c	Race	**Race-stratified analyses:** For African American women, greater everyday discrimination was associated with CIMT after adjustment for age; this association was reduced to marginal significance after adjustment for HDL-c and BMI. -No associations were found for White women. -No associations were found between CIMT and attribution to race/ethnicity for everyday discrimination
Endothelin- 1	Cooper et al. (2009) [103]	Black (*n* = 51) and White (*n* = 65) participants from a larger cardiovascular study between 2000–2005, San Diego, CA. *N* = 116, mean age = 36.5	Cross-sectional	Ethnic discrimination	Gender*, exercise, BMI, resting mean arterial pressure, and social desirability	–	Race	**Race stratification/race interaction:** For Black participants, greater discrimination was associated with greater endothelin- 1 dysfunction. No associations were found for White participants
White matter hyperintensity (WMH) and hippocampal hyperintensity volume (assessed via brain MRI)	Zahodne et al. (2023) [104]	Non-Hispanic Black participants (US-born: *n* = 204, Foreign-born: *n* = 17) from the Washington Heights-Inwood Columbia Aging Project (WHICAP), 2011–2017. Northern Manhattan, NY.* N* = 221, age = 65 and older; mean age = 73.46	Longitudinal	-Everyday discrimination -Lifetime discrimination	Age, sex/gender*, education, income, time between scans, and total intracranial volume at the initial scan	–	–	-Greater lifetime racial discrimination was associated with lower initial hippocampal volume. -Greater everyday racial discrimination, but not lifetime racial discrimination, was associated with faster accumulation of WMH over time - No predictive associations between aggregate everyday or lifetime discrimination measures and initial hippocampal or WMH volume or changes in hippocampal or WMH volume
White matter lesion volume (WMLV; assessed via brain MRI)	Beatty Moody et al. (2019) [51]	African American participants from HANDLS, MRI Ancillary Project, 2004–2009. Baltimore, MD. *N* = 71, age range = 33–69, mean age = 50	Cross-sectional	-Racial discrimination -Lifetime discrimination burden	-Age, sex*, and SES composite (education and poverty status) -Sensitivity analyses with BMI, HTN, diabetes, total cholesterol, cigarette and alcohol use, waist circumference (WC), and depressive symptoms (CES-D) -Additional analyses excluded CVD or kidney disease history and poverty status	–	Age	**Age interaction:** In older African American adults (i.e., 60 years old), greater discrimination (both measures) were associated with greater WMLV.—In younger African American adults (i.e., 40 years old), less racial discrimination was associated with greater WMLV, and no association for lifetime discrimination was found
C) Individual-Level Discrimination and Racism and Self-Reported Subclinical and Clinical CVD								
Arteriosclerosis, MI, and other'minor'heart diseases (i.e., angina pectoris, tachycardia) (Yes/No to each; self-reported)	Udo & Grilo (2017) [105]	Black (non-Hispanic) (*n* = 5398), White (non-Hispanic) (*n* = 15.520), and Hispanic (*n* = 5,101) women from the National Epidemiologic Survey on Alcohol and Related Conditions (NESARC), 2001–2002 and 2004–2005. Multi-state sample. *N* = 26,992, mean age = 49.2	Cross-sectional	Racial discrimination	Age, gender*, race/ethnicity, income, education, marital status, cigarette and alcohol use, BMI, DSM-IV diagnosis, and stressful life events	–	–	**Full sample analyses:** Greater discrimination was associated with greater odds of 3-year incidence of CVD (self-reported arteriosclerosis, MI, and minor heart conditions) in surveyed women
Atherosclerosis, MI, stroke, or other heart problem, blood circulation problem, and/or high cholesterol (total count and Yes/No to any; self-reported)	Cuevas et al. (2019) [106]	Black (*n* = 586) and White (*n* = 520) participants from the Detroit Area Study (DAS), 1995. Detroit, MI.* N* = 1,106, age range = 18–97	Cross-sectional	Racial discrimination lifetime exposure (If yes, age at time of first experience)	Age, gender*, race, education, income, marital status, physical activity, smoking, and self-reported pre-existing health conditions	–	-Race -Gender*	**Full sample analyses:** For Black participants, greater discrimination was associated with greater self-reported CVD risk. No significant interactions with race or gender were found
Atherosclerosis, MI, and/or stroke (self-reported)	Chae et al. (2012) [107]	Black American participants from the National Survey of American Life (NSAL) study, 2001–2003. Multi-state sample. *N* = 5,022, mean age = 36.4	Cross-sectional	Racial discrimination	Age, sex*, ancestry/ethnicity, education, poverty level, employment status, insurance status, marital status, physical activity, smoking status, BMI, number of chronic conditions from a checklist of 14 common health issues (excluding cardiovascular outcomes), region of residence, and social desirability	–	Mood disorder history (i.e., major depression, dysthymia, and/or bipolar disorder) as assessed on the World Mental Health Survey Initiative version of the WMH-CIDI	**Full sample analyses:** For Black Americans, greater discrimination among those with a mood disorder history was associated with a greater risk of self-reported CVD, with this effect being especially pronounced in younger Black American adults (< 50). No significant associations were found between discrimination and self-reported CVD for Black Americans without a mood disorder history
Atherosclerosis, MI, and/or stroke (self-reported)	Chae et al. (2010) [108]	African American men from the National Survey of American Life (NSAL), 2001–2003. Multi-state sample. *N* = 1,216, mean age = 41.8	Cross-sectional	Racial discrimination	Age, education, poverty level, employment status, insurance status, marital status, smoking status, BMI, region of residence, and social desirability	–	Negative African American racial group attitude (e.g., agree/disagree that Blacks are lazy, give up easily, and/or violent)	**Full sample analyses:** For African American men, greater discrimination among those with negative racial group beliefs about African Americans was associated with greater risk of self-reported CVD. However, no significant direct association between discrimination and self-reported CVD was found
Cardiovascular conditions (e.g., hypertension, stroke, blood circulation problems, and heart problems or heart attack) (self-reported)	Mouzon et al. (2017) [109]	African American participants from the National Survey of American Life (NSAL), 2001–2003. Multi-state sample. *N* = 3,570. Age range = 18- 93, mean age = 42	Cross-sectional	Everyday discrimination	Age, gender*, education (in years), family income, employment status, marital status, and region of residence	–	–	**Full sample analyses:** For African Americans, greater everyday discrimination was associated with greater odds of all types of health problems, including self-reported CVD
Cardiovascular health (e.g., heart attack, stroke, heart disease, high blood pressure, and blood circulation) (self-reported)	Carlisle et al. (2015) [110]	Native and foreign-born Asian American (*n* = 1,628), Latino-American (*n* = 1,940), and Afro-Caribbean American (*n* = 1,438) participants of the Collaborative Psychiatric Epidemiology Surveys (CPES), including the National Latino and Asian American Study [NLAAS], 2002–2004, and NSAL, 2001–2003. Multisite sample. *N* = 5,006, Age range = 18–65	Cross-sectional	Perceived discrimination	Age, gender*, education, income, and marital status		-Nativity -Length of residency	**Full sample analyses:** Afro-Caribbean subgroups were more likely to report perceived discrimination at all three levels (low, moderate, and high) than Asian American and Latino-American subgroups - No association was found between perceived discrimination and CV health
Chest pain/angina and/or rapid heartbeat/tachycardia (self-reported)	Gavin et al. (2021) [111]	Non-Hispanic Black adult participants of the National Epidemiologic Survey on Alcohol and Related Conditions-III (NESARC-III) Study, 2013. Multi-state sample. *N* = 7,766, mean age = 43.4	Cross-sectional	Racial discrimination	Age, education, income, marital status, nativity, and major depressive disorder and/or substance use disorder (measured using AUDADIS- 5)	PTSD	–	**Full sample analyses:** For Black adults, greater discrimination was associated with greater odds of all CVD outcomes (e.g., chest pain/angina and tachycardia). PTSD symptoms mediated these associations in this population
Hypertension, atherosclerosis, stroke, or MI (self-reported)	Whaley (2022) [112]	African Americans (*N* = 3,570), which includes Afro-Caribbean (*n* = 1419) participants. Afro-Caribbean subsample further divided by nativity: US-born (*n* = 373) and Foreign-born (*n* = 373). National Survey of American Life (NSAL), 2001–2003. Multisite sample. Mean age = 42.26	Cross-sectional	-Everyday discrimination -Negative racial stereotypes -Perceived mastery (control and competence over one's life)		–		-***African American subsample analyses***: Positive associations were observed between negative racial stereotypes and perceived discrimination and CVD (all types), with the latter having a stronger effect -***US-born African Caribbean subsample***: Positive association was observed between negative racial stereotypes and CVD (all types) -***Foreign-born African Caribbean subsample***: Positive association was observed for negative racial stereotypes with high blood pressure only
D) Cultural-Level Discrimination and Racism and Subclinical and Clinical CVD								
- CVD (i.e., HTN, diabetes, obesity, stroke, MI, and/or CHD) (self-reported) -Any CVD, defined as 1 or more CVD events (i.e., stroke, MI, CHD)	Huang et al. (2020) [64]	African American, White, Multiracial, Hispanic, and Other Participants** from the Behavioral Risk Factor Surveillance System (BRFSS), 2017. Data collection from the Twitter Streaming Application Programming Interface, 2015–2018. All US states and District of Columbia. Multisite sample. *N* = 433,434 to 433,680 across outcomes	Ecological (cross-sectional)	-Twitter-characterized sentiment towards racial/ethnic minorities (30 million race-related tweets collected via Twitter Streaming Application Programming Interface from 2015–2018) -Prevalence of negative sentiments of race related tweets towards racial/ethnic minorities calculated at state level	-State-Level: % non-Hispanic White, % non-Hispanic Black, % Hispanic, and median household income from 2017 American Community Survey (1-year estimate) -Individual-level: age, sex*, race/ethnicity, education, and marital status from BRFSS		Race	-**Full sample analyses:** Greater negative sentiment associated with greater stroke, MI, CHD, and any CVD risk -**Race-stratified analyses:** Greater negative sentiment associated with greater HTN, MI, and any CVD risk, with a more pronounced effect in non-Hispanic Black and White participants
CVD mortality (e.g., chronic rheumatic heart disease, hypertensive diseases, ischemic heart diseases, pulmonary heart disease, and other forms of heart diseases)	Zestcott et al. (2022) [113]	Black, Native American, and White participants from Project Implicit (2003–2015) and CDC Wonder Database, 2003–2015 Implicit prejudice (*N* = 1,946,209): Whites (*n* = 1,612,211), Blacks (*n* = 319,434) and Native Americans (*n* = 14,564). Explicit prejudice (*N* = 2,037,623): Whites (*n* = 1,681,876), Blacks (*n* = 340,943), and Native Americans (*n* = 14,804). Data averaged per each county and state. Number of counties varied for each racial group (*n* = 697 for Whites, *n* = 636 counties for Blacks, and *n* = 214 for Native Americans)	Ecological (cross-sectional)	Implicit and explicit prejudice	Age, population, education, income, employment status, and health insurance status	–	–	**Overall:** Across Black, Native American, and White racial groups, racial prejudice is associated with CVD mortality risk for both the prejudiced and the stigmatized groups **At the state level:** Whites'explicit prejudice toward Blacks and implicit prejudice toward Native Americans were positively associated with greater CVD mortality for Whites **At the county level:** -Whites'implicit prejudice toward Blacks and Blacks'implicit prejudice toward Whites were positively associated with greater CVD mortality for Whites -Blacks'implicit and explicit prejudice toward Whites were associated with CVD mortality for Blacks -Whites'explicit prejudice was positively associated with Blacks'CVD mortality
Circulatory-disease risk (access to health care, diagnosis of a circulatory disease) and circulatory-disease related death rate	Leitner et al. (2016) [114]	Black (*n* = 250,665) and White (*n* = 1,391,632) participants from Project Implicit, 2003–2013. Death records were obtained from the CDC (2014). United States (excluding territories and foreign military bases). Multisite sample.* N* = 1,642,297	Ecological (cross-sectional)	Whites’ explicit or implicit racial bias	Age, sex*, population, education, income, unemployment, and poverty, segregation, geographic mobility, housing density, and age bias	–	Race	-Black-White health disparities in circulatory disease risk and circulatory-disease-related mortality rate were more pronounced in communities where Whites harbored more explicit racial bias -The positive relationship between explicit racial bias and circulatory-disease-related mortality was significant for both groups, but the relationship was stronger for Black than White participants
Heart attack and stroke-specific mortality rates	Chae et al. (2015) [115]	Black and White Americans** in designated market areas (DMAs) in the United States (*n* = 196). Mortality outcomes data for black Americans aged 25 and older. National Center for Health Statistics, 2004–2009. Multisite sample	Ecological (cross-sectional)	Area racism: defined as % total Internet search queries containing the “N-word” [singular or plural, 2004–2007] per Google searches within designated market areas	-Designated market area-level: urbanicity, % African Americans, education, and poverty among African Americans; White mortality rate and all cause-specific mortality rates; African American mortality rates from HD, cancer, stroke, and diabetes -Individual level: decedent’s age, sex*, year of death, and US Census region of residence (per death certificates)	–	–	**Full sample analyses:** Greater area racism was associated with higher heart attack and stroke-specific mortality rates in Black Americans
Heart failure prevalence and stroke prevalence	Splan et al. (2021) [116]	Black and White American patients aggregated to county level.** Project Implicit, 2002–2017. *N* = 2.45 million implicit and explicit thermometer ratings from *N* = 836 counties. Multisite sample. Medicare recipients aged ≥ 65	Ecological (cross-sectional)	Implicit and explicit racial attitudes	Age (median age), sex* (gender ratio), education (White and Black high school completion rates), White and Black poverty rates, county-level assault rates, population density, White and Black population percentages, net migration, political preferences, and religiosity		Race	-Black and White patients who live in areas with high implicit and explicit racial bias tend to exhibit a higher incidence of chronic health problems, including heart failure and stroke. These relationships were stronger for Black patients -Patients in racially diverse and racially segregated regions have a higher incidence of chronic health problems, including heart failure and stroke
E) Structural- and Institutional-Level Discrimination and Racism and Subclinical and Clinical CVD								
CHD and/or stroke (self-reported)	Nardone et al. (2020) [117]	US Census Tracts (*N* = 4,061) from 9 cities (Atlanta, GA; Chicago, IL; Cleveland, OH; Los Angeles, CA; Miami, Fl; New York, NY; Oakland, CA; San Francisco, CA; and St. Louis, MI). Health outcome data from Centers for Disease Control and Prevention 500 cities program, 2010 Census	Ecological	HOLC color-coded security denoting lending risk: -Green (best/least financial risk) - Blue (still desirable) -Yellow (declining) -Red (hazardous/most financial risk) -Redlined neighborhoods shaded red	No adjustments	–	–	**Full sample analyses:** Historically redlined areas were associated with greater stroke risk in 5 of 9 cities and with greater risk of CHD in 4 of 9 cities
CVD-related mortality	Kramer et al. (2017) [118]	Black and White populations from counties (*N* = 1,100 in 1860, *N* = 1,347 in 2010) where slavery was legal in 1860 (source: the National Center for Health Statistics). Multi-state sample. Temporal patterns assessed for 1968–2014	Ecological	Percent (%) population enslaved (per 1860 U.S. Census)	Size and diversity of county economies (average value farmland and buildings per acre and annual economic output from manufacturing [from 1860]); Black-White disparities in illiteracy (from 1930), college attainment (from 1970), unemployment and median home value (from 1930), and poverty rates (from 1970); and the number of lynchings in each county (between 1877–1950)	–	–	**Race-stratified analyses only:** Black populations from counties with greater historical enslavement concentrations experienced slower declines in CVD-related mortality. No significant association was found between slave concentration and CVD mortality declines for White populations
Major adverse CV events (MACE) (e.g., myocardial infarction, stroke, major adverse extremity events, and all-cause mortality)	Deo et al. (2023) [119]	Black (*n* = 29,873), Asian (*n* = 463), Hispanic (*n* = 4,341), American Indian or Alaska Native (*n* = 914), White (44,584), and Unknown (*n* = 840) Veterans from the Strengthening the reporting of observational studies in epidemiology (STROBE) Cohort Study. Data collected 2016–2019, Analysis performed 2022. HOLC Coverage Areas (200 US cities). *N* = 79,997, mean age = 74.46	Retrospective cohort	Redlining (defined as D-graded neighborhoods based on HOLC's grade of the Census tracts of residence)	Age at index visit, sex*, self-reported race (American Indian or Alaska Native, Asian, Black, White, declined to report, and unknown) and ethnicity (Hispanic, Not Hispanic, and unknown), smoking status, hypertension, presence of diabetes, chronic kidney disease, atrial fibrillation, and history of myocardial infarction (MI) or percutaneous coronary intervention	–	–	Living in a historically redlined neighborhood was associated with a 14% higher risk of the composite MACE outcome and a 15% higher risk of MI. This risk was lower, yet remained significant, after adjusting for social vulnerability and comorbidity burden
MI (self-reported in the past year)	Lukachko et al. (2013) [120]	Black (non-Hispanic) (*n* = 8,245) and White (non-Hispanic) (*n* = 24,507) participants of the National Epidemiologic Survey on Alcohol and Related Conditions (NESARC), 2001–2002. Multi-state sample *N* = 32,752, age 18 and over	Ecological (cross-sectional)	Structural racism in four domains: (1) Political participation (2) Employment and job status (3) Educational attainment (4) Judicial treatment	Age, sex*, education, household income, medical insurance, and state-level racial disparities in poverty	–	Race	-**Race-stratified analyses:** For Black participants, high levels of structural racism in 2 of the 4 domains (political participation & judicial treatment) were associated with increased MI incidence. High levels of structural racism in employment and job status was associated with lower MI incidence. No associations were observed between structural racism in educational attainment and MI incidence for Black participants. -For White participants, high structural racism in political participation & judicial treatment were associated with decreased MI. No associations were observed between structural racism in employment and job status or educational attainment and MI incidence for White participants -**Race interaction analyses:** 3 of 4 domains showed significant racial differences—political participation and judicial treatment domains were associated with increased MI incidence for Black participants and lower MI incidence for White participants. Job status was associated with lower MI incidence in Black participants but not associated with MI for White participants

Mean age provided when available

^*^Binary measure of biological sex (Male/Female)

^**^Study does not report information on race/ethnic subpopulations

^***^Study does not report age

List of Abbreviations: *African American* African Americans, *AUDADIS-V* Alcohol Use Disorder and Associated Disabilities Interview Schedule Associated Disabilities Interview Schedule, *DSM-V* Version, *BDI* Beck Depression Inventory, *BMI* Body Mass Index, *CAC* Coronary Artery Calcification, *CES-D* Center for Epidemiologic Studies Depression Scale, *CHD* Coronary Heart Disease, *CI* Confidence Interval, *CIMT* Carotid Intimal-Medial Thickness, *CRP* C-reactive Protein, *CVD* Cardiovascular Disease, *DBP* Diastolic Blood Pressure, *EGFR* Estimated Glomerular Filtration Rate, *FRS* Framingham Risk Score, *HD* Heart Disease, *HDL-C* High-density Lipoprotein Cholesterol, *HOLC* Home Owners Loan Corporation, *HTN* Hypertension, *LDL-C* Low-density Lipoprotein Cholesterol, *LVH* Left Ventricular Hypertrophy, *LOT-R* Revised Life Orientation Test, *MI* Myocardial Infarction, *MRI* Magnetic Resonance Imaging, *NEO PI-R* NEO Personality Inventory-Revised, *PEDQ* Perceived Ethnic Discrimination Questionnaire, *SBP* Systolic Blood Pressure, *SES* Socioeconomic Status, *WC* Waist Circumference, *WMH* White matter hyperintensity, *WMH-CIDI* World Health Organization Composite International Diagnostic Interview, *WMH* White matter hyperintensity, *WMLV* White Matter Lesion Volume

### Structural- and Institutional-Level Discrimination and Racism and Subclinical and Clinical CVD

With regard to structural-level discrimination and CVD, we identified four studies [117–120] (see Table 2) focusing on broad markers of racism within or across institutions. Kramer et al. [118] investigated whether county-specific slavery legacy (i.e., counties in states where slavery was legal in 1860) —a historic institution of racism—was associated with the temporal and geographic patterning of heart disease mortality in Blacks and Whites in the U.S. They observed that Black populations in counties with the highest historical concentrations of slavery (per 1860 U.S. Census data), experienced a 17% slower decline in heart disease mortality from 1968 to 2014, compared to counties with the lowest historical concentrations. The association for Black populations varied by region and was partially explained by intervening socioeconomic factors, as well as the cumulative use of lynchings to enforce Black subordination. No association was observed between slave concentration and heart disease mortality decline among Whites.

Nardone et al. [117] demonstrated associations between another historical indicator of institutional racism and self-reported stroke and CHD. Specifically, the authors linked residential redlining, via the 1930’s government-established Home Owners’ Loan Corporation Security Maps, which categorized neighborhoods with high concentrations of Blacks as ‘‘high risk,’’ to present-day health outcome data from the 500 Cities data set (via the Behavioral Risk Factor Surveillance System, CDC) across 9 major U.S. cities. They obtained additional data from the American Community Survey, which showed lower present-day median incomes, along with a higher percentage of people of color, in historically redlined neighborhood. Redlined areas were associated with increased prevalence of CHD in 4 of the 9 cities, and with increased prevalence of stroke in 5 of the 9 cities. Similarly, Deo et al. [119] demonstrated that residing in historically redlined neighborhoods was associated with a 14% higher risk of a major adverse cardiovascular event in a cohort study of 79,997 U.S. veterans being treated for atherosclerotic disease. Associations were independent of traditional cardiovascular risk factors. No association was noted between area of residence and stroke.

There is also evidence of adverse linkages between more contemporary indicators of structural racism and clinical CVD. Lukachko et al. [120] showed that multiple U.S. state-level factors examined between 2000 and 2005, including political participation, employment status, educational attainment, and judicial treatment, were associated with racial disparities in past-year myocardial infarction (MI) incidence. Independent of individual-level confounders and state-level disparities in poverty, Blacks living in states with high levels of structural racism were generally more likely to report past-year MI than Blacks living in low-structural racism states. Conversely, Whites living in high structural racism states experienced null or lower odds of MI compared to Whites living in low-structural racism states, raising the provocative possibility that structural racism may not only harm the targets of stigma but benefit those who hold power.

### Cultural-Level Discrimination and Racism and Subclinical and Clinical CVD

We identified five studies [64, 113–116] (see Table 2) that utilized novel indicators of cultural racism in examining potential links with CVD. Chae et al. [115] used an internet search-based proxy for geographical racism and observed racial disparities in all-cause and cardiovascular-specific mortality (heart attack and stroke) in Blacks. Specifically, area racism was defined as the proportion of Google searches containing the “N-word” in 196 designated market areas (DMAs); these searches were then examined in relation to mortality data from the National Center for Health Statistics (2004–2009). DMAs characterized by a one standard deviation greater level of area racism were associated with an 8.2% increase in the all-cause Black mortality rate, equivalent to over 30,000 deaths annually. This effect was slightly attenuated after adjustment for DMA-level demographic and socioeconomic covariates (e.g., percent of Black households living in poverty). Further, area racism was significantly associated with Black mortality rates for heart disease and stroke.

A second study analyzed Twitter-derived sentiments towards racial/ethnic minorities [64], categorized as “positive” or “negative,” drawn from 30 million tweets from 2015 to 2018. Independent of individual- and state-level demographics, respondents living in states with the highest level of negative sentiment towards racial/ethnic minorities had a 30% higher prevalence of stroke, 14% higher prevalence of MI, 9% higher prevalence of CHD, and 16% higher prevalence of *any* CVD outcome. In-race stratified analyses, the relations of negative racial sentiment to MI and any CVD outcome were stronger in non-Hispanic Blacks and Whites than in those identifying as Hispanic, multiracial, or “other race,” but were not significant (although confidence intervals included the null in all cases).

Three other studies [113, 114, 116] leveraged data from Project Implicit, a website maintained by an international network of researchers (https://www.projectimplicit.net/) that houses measures of explicit and implicit bias. In these studies, explicit bias was measured by respondents’ direct reports of how warm they feel toward their own, as well as other, racial groups, while implicit bias was measured with the Implicit Association Test. The findings in these studies generally suggest that both holding, and being the target of, racial prejudice may have detrimental effects on cardiovascular health. Specifically, in the Leitner et al. [114] and Splan et al. [116] studies, *both* Blacks and Whites residing in regions characterized by greater racial bias among Whites were at increased risk for death due to circulatory disease [114] or heart failure and stroke [116], with generally stronger effects for Blacks. Somewhat similarly, Zestcott et al. [113] reported that holding higher levels of prejudice toward the other racial group was associated with increased CVD mortality in a study that included both Black and White adults. However, these findings were more complex, in that associations between bias and CVD mortality depended on state versus county-level analysis, as well as the form of bias under study (generally, harboring explicit bias was more predictive of CVD at the state level, while harboring implicit bias was more predictive at the county-level). One limitation consistently noted by the authors of these studies is the non-representativeness of the sample; participants visiting the Project Implicit website are self-selected and tend to differ from the larger population on key demographic variables, such as age.

### Summary: Structural-, Institutional-, and Cultural-Level Discrimination and Racism and Subclinical and Clinical CVD

In summary, these nine studies provide novel and compelling support for the adverse impact of structural, institutional, and cultural-level indicators of racism and suggest that the historical imprint of racist institutions (e.g., the enslavement of Blacks; neighborhood redlining), and the subsequent legacy of these systems (e.g., continuing cultural bias), influence racialized patterns in cardiovascular morbidity and mortality. Endpoints focused largely on major cardiovascular events or mortality, with most studies utilizing health records [113–116, 118, 119], and three studies relying on self-report [64, 117, 120]. When possible, directly and comprehensively assessing a variety of clinical outcomes will help clarify which forms of CVD are most consistently linked to structural and cultural racism. In addition to clinical outcomes, examining these exposures in relation to the progression of subclinical CVD indicators over time is necessary to establish temporal and putatively causal relationships and elucidate the biological pathways through which discrimination exerts harmful effects.

The conceptualization and measurement of racism varies widely, with exposures including historical racist practices, ‘proxy’ indicators of Black/White disparities (i.e., educational attainment), and digital communications. These commendable studies highlight some of the methodological complexities of work on the systemic influences of cardiovascular risk. Others [121, 122] also note a larger need for guidance and standardization related to the conceptualization and quantification of cultural, institutional, and structural-level markers of racism. Moreover, despite the large body of evidence demonstrating racial inequities in healthcare [123]*,* few studies examine parallel markers of structural racism specific to the healthcare setting and their potential impact on cardiovascular health outcomes. Developing measures that capture institutional and cultural inequalities faced by African Americans and other racial/ethnic minorities specific to healthcare domains is an important step in understanding how these systems shape race-based inequities in cardiovascular health.

Lastly, select data indicate that the relation of structural racism to CVD may, in part, be mediated by socioeconomic factors [115, 117, 118]. Historical and contemporary discriminatory practices have created and perpetuated systematic socioeconomic disadvantages, including disparities in wealth, housing, and neighborhood conditions for African Americans in the U.S. These disadvantages are, in turn, associated with increased cardiovascular morbidity and mortality [124]. When studies include mediators in their model – without recognizing them as mediators – the potential for type 2 error occurs. We might “overcontrol” the model and fail to recognize or underestimate an association between discrimination and CVD. Thus, future work should more formally consider which factors are potential mediators and explicitly test for such mediation. Such factors are likely accompanied by myriad individual-level factors (e.g., chronic stress) and compounded by interpersonal experiences to further undermine cardiovascular health. These individual-level experiences are discussed next.

### Individual- or Interpersonal-Level Discrimination and Racism and Subclinical and Clinical CVD

Studies of individual-level discrimination, which make up the bulk of the literature examining discrimination and CVD, are shown in Table 2. The majority of this work is cross-sectional, with *everyday* and *racial* discrimination as the primary predictors of interest. A handful of additional studies also captured lifetime or chronic burden of discrimination, or assessed expectations of future treatment. Below, we review highlights of this work, clustered by type of outcome (measured clinical endpoints, measured subclinical risk, and self-reported outcomes).

Six studies [27, 87–91] of interpersonal discrimination included a *measured* clinical CVD endpoint. These outcomes include incident stroke, coronary obstruction, and composite measures of cardiovascular mortality (shown in Section A of Table 2). The one study that employed a cross-sectional design reported increased cardiovascular risk of severe coronary obstruction associated with discrimination in Black but not White participants [27]. The other five studies were longitudinal, with follow-up periods ranging from 8 to 20 years. In the first, which included 48,924 African American women, there was no association between everyday discrimination and CVD-specific death across an 8-year follow-up [88]. In the second, everyday and lifetime discrimination were not associated with incident CHD or incident stroke across ~ 9 year follow-up in older African Americans in the Jackson Heart Study [89]. In the third investigation, Black women reporting that they had experienced racism in three separate domains (employment, housing, and interactions with police) had an increased risk of stroke over 20 years later [91]. Finally, the fourth and fifth studies were based on longitudinal reports from the Multi-Ethnic Study of Atherosclerosis, and considered exposure to both lifetime and everyday discrimination. In the fourth study, Everson-Rose et al. [90] reported that individuals endorsing more lifetime or everyday discrimination were at increased risk for incident CVD across ~ 10 year follow-up; the risk was attenuated after adjusting for stress and depressive symptoms. In the fifth study [87], both lifetime discrimination and everyday discrimination were associated with increased cardiovascular mortality nearly 20 years later. The risk associated with lifetime versus everyday discrimination differed by race in stratified analyses, however, with lifetime discrimination predicting CVD mortality in Black participants and everyday discrimination predicting CVD mortality in White participants.

We identified 14 studies [51, 92–104] that included a measured (versus self-reported) indicator of subclinical CVD risk, such as carotid intima-media thickness (CIMT), coronary artery calcification, arterial stiffness, endothelin- 1, or cerebral white matter lesion volume (an indicator of subclinical CeD) (shown in Section B of Table 2). Of the three that were longitudinal in design, one reported a link between greater everyday racial discrimination and faster accumulation of white matter lesions over time in a sample of older African Americans [104], another reported a link between greater *chronic* everyday discrimination over five years and the presence of coronary artery calcification in African American women [96], and the third reported a link between higher levels of everyday discrimination and greater CIMT in White women only [101]. Of the eleven cross-sectional reports, all but one [95] reported a link between discrimination and cardiovascular risk. However, several of these associations were qualified by psychosocial [94, 98] or sociodemographic [51, 92] moderators, or were reduced to marginal significance after adjusting for traditional risk factors [102]. Of note, three of these studies reporting significant links observed *inverse* associations, such that lower levels of self-reported discrimination were related to increased risk, either in the full sample [93, 100] or in a sample sub-groups (e.g., younger African Americans) [51].

Eight additional studies [105–112] included only self-reported cardiovascular endpoints (shown in Section C of Table 2). These studies were predominately conducted in exclusively African American or Black samples (i.e., [106–109, 111, 112]), and most used racial discrimination as the main exposure variable. In the one available longitudinal study, reporting racial discrimination was related to increased incidence of arteriosclerosis, MI, and minor heart conditions over 3-year follow-up, after adjustment for sociodemographic, health, and psychosocial factors, in 26,992 African American, White, and Hispanic respondents [105]. Of the remaining seven cross-sectional studies, six reported that higher levels of self-reported discrimination were associated with greater odds of poorer cardiovascular health, including endpoints such as chest pain, angina, atherosclerosis, MI, and stroke. Notably, most studies took a count of cardiovascular-related endpoints, grouping together multiple (e.g., stroke or MI) outcomes. In the one study reporting null results, reports of discrimination were not associated with self-reported cardiovascular conditions in a multi-ethnic sample of Black Caribbean immigrants (Haitian, Jamaican, and Trinidadian/Tobagonian), although adjusting for discrimination somewhat attenuated the heightened cardiovascular risk in Trinidadians relative to Jamaicans (the reference group for analysis) [110].

### Summary: Individual- or Interpersonal-Level Discrimination and Racism and Subclinical and Clinical CVD

In summary, 15 of 19 cross-sectional studies and 6 of 9 longitudinal studies suggest that greater self-reported exposure to discrimination or racism is associated with increased risk for clinical or subclinical forms of CVD in African Americans or in samples that include African Americans. Interestingly, three cross-sectional studies [51, 93, 100] observed greater cardiovascular risk among African Americans reporting low levels of discrimination, suggesting that the relation may be nonlinear. These counterintuitive findings may possibly reflect the health-protective effects of acknowledging racial discrimination or suggest that the impact of experiencing racism on health is conditional on other psychological processes, such as coping, internalized racism, or locus of control [51]. Moreover, there is some evidence from these and other studies (e.g., [48]) that age may moderate the nonlinear association between discrimination and cardiovascular risk, with inverse links more likely to be observed among younger samples.

While it is established that interpersonal discrimination and racism are more commonly reported in African Americans than White adults, 6 of the 13 studies that included both Black and White adults suggest that the negative cardiovascular impacts of discrimination may be stronger in African Americans [27, 87, 92, 98, 102, 103]. In contrast, one study of everyday discrimination showed deleterious effects in White women but not Black, Chinese, or Hispanic women [101]. In that study, the most common reason for unfair treatment reported by White women was “gender,” reflecting the multi-faceted and likely cumulative nature of discrimination (also see weight discrimination in [105]). In a similar vein, 8 of the 28 studies reported that links between discrimination and cardiovascular health were conditional upon sociodemographic variables (e.g., gender, age), other identity statuses (e.g., immigration) or individual-difference factors (e.g. depressive symptoms, coping style, attitude toward African Americans). Thus, a comprehensive understanding of the ways in which discrimination influences cardiovascular health will require consideration of the cumulative and interacting effects of various forms of discrimination, along with person-level factors such as psychosocial and mental health.

The mediating pathways linking discrimination to cardiovascular outcomes are likely multiple and complex. Each of the 20 studies that included a measured cardiovascular endpoint adjusted for health factors or behaviors that may be on the mediational pathway, such as body mass index (BMI), blood pressure, physical activity, and/or smoking. These factors were less consistently measured in studies that focused on self-reported outcomes, with just 4 of the 8 including BMI or health behaviors as covariates [105–108]. With regard to psychosocial factors, 11 of the 28 studies assessed variables such as chronic stress, depressive symptoms, or negative affect [27, 51, 89–93, 98, 99, 105, 111]. However, few studies performed formal tests of mediation. One cross-sectional study [102] demonstrated partial mediation of CIMT by traditional cardiovascular risk factors, and one longitudinal study [90] demonstrated partial mediation of incident CVD by stress and depressive symptoms. In the next section, we discuss several of the most promising biological and psychosocial mechanisms linking discrimination to CVD outcomes.

## Biopsychosocial Mechanisms: How Might Multilevel Racism and Discrimination Be Adversely Related to Cardiovascular Health?

In this section, we identify pathways that may connect experiences of discrimination and racism to cardiovascular morbidity and mortality. We again use a multilevel conceptualization of discrimination, as we consider distal, intermediate, and proximal mediators as identified in previous research [125].

At the distal level, a complex array of societal factors may overlap and interact with discrimination and racism to maintain environments with limited resources and heightened cardiovascular risk. In their recent review, Juarez et al. [126] describe how African Americans are exposed to a multitude of environmental CVD risk factors at higher rates than Whites. In this regard, Powell-Wiley et al. [15] in their review of social determinants of CVD detail macro to micro socioenvironmental contexts that bare upon CVD processes and outcomes. Such factors may occur in the natural environment (e.g., pollution, heavy metals, and pesticides), the built environment (e.g., neighborhood conditions and accessibility of healthy food), and/or the social environment (e.g., access to healthcare services, quality of patient-provider interactions, and population density). These environmental conditions persist over time via racial segregation and political fragmentation, which are subsequently correlated with poor economic outcomes [127]. Disinvested communities then have fewer resources to help buffer against negative health outcomes [128, 129]. Accessing healthcare is also more difficult, which may exacerbate feelings of mistrust of the medical community and further sustain healthcare-related inequalities [130–134]. Recently, several groups have published comprehensive reports offering a variety of interpersonal, institutional, and policy-level interventions aimed at reducing these more distal risk factors for racial health disparities [135–137], and have argued for the expansion of existing social determinants of health frameworks to incorporate the roles of social exclusion and marginalization to more accurately reflect the experiences of minority groups [15].

Considered traditional CVD risk factors, as well as diseases in their own right, hypertension, diabetes, and obesity are key intermediaries of the association between discrimination and CVD outcomes (e.g., [138]). Evidence of these associations exist at both the institutional and interpersonal levels of discrimination. For example, a review by Brondolo et al. [24] linked institutional racism, in the forms of residential racial segregation and incarceration, to hypertension incidence, while a 2014 review concluded that racial discrimination is related to hypertension, especially when measured via nighttime ambulatory monitoring [82]. Regarding markers of adiposity, most work has focused on interpersonal discrimination. Relationships are observed in many, but not all, studies, with some data showing that these links may be stronger among women than men (e.g., [139]). In addition, a recent review [140] outlined the role of systemic racism in Black, indigenous, and other people of color with respect to increased rates of obesity. Fewer studies have examined discrimination and diabetes; but women reporting the highest levels of racism in the Black Women’s Health Study were more likely to develop diabetes, partially due to higher BMI [141].

At the most proximal level, discrimination may serve as a direct form of psychosocial stress with behavioral and psychological consequences. Even in the absence of immediate unfair treatment, past experiences, as well as anticipation of future ones, can leave a residue that chronically “taxes” one’s cognitive, emotional, and behavioral resources. Further, discrimination and racism are often uncontrollable and unpredictable, making them particularly potent sources of stress, especially when appraised as threatening or as exceeding one’s coping resources (e.g., [29, 142, 143]). As such, discrimination has been linked to maladaptive lifestyle and behavioral changes (e.g., [144]), such as disturbed sleep, increased substance use, and increased fat consumption [145], all of which contribute to increased disease risk. Experiences of discrimination have also been associated with shifts in psychological and mental well-being (e.g., [146], including increases in depressive, anxiety, vigilance, and trauma symptomology [147, 148].

Stressful experiences of discrimination, along with their impact on behavioral and psychological factors, may subsequently impair the body's ability to regulate key biological systems, resulting in physiological alterations [25, 28, 149–151] that, over time, contribute to disease risk. For example, discrimination is related to a higher allostatic load, or dysregulation in physiological mechanisms due to chronic stress [152], with key candidate pathways including changes in neuroendocrine, immunologic, and cardiovascular activity [25, 151]. Interestingly, exposing African Americans and other minorities to experiences of discrimination in experimental settings results in aberrations in these physiological systems (e.g., [153]), supporting a potential mediational role in the progression of CVD.

Additional mechanisms may lie at the cellular and neurobiological levels [154]. Empirical studies of everyday and racial discrimination have been linked to measures of increased oxidative stress [155], shortened telomere length [156], and DNA methylation [157]. Epigenetic models in which the stress of discrimination may act cross-generationally through the impact of early life environmental exposure on gene expression also have been posited [158, 159], offering a framework for examining how distal and proximal factors may interact to increase cardiovascular risk. Recent reviews also highlight the role of neurobiological systems in the discrimination-CVD relationship, theorizing that differential patterns of neural functioning in response to experiencing racism result in changes in peripheral physiology, including increased sympathetic, HPA, and pro-inflammatory activity [25, 160].

In sum, many plausible biopsychosocial mechanisms, spanning multiple levels of discrimination, have been identified on the complex explanatory pathways to CVD. But relatively little work has directly examined these mechanisms in the context of a multi-level conceptualization of discrimination, or in relation to intersectional identities. For instance, individuals with intersecting marginalized identities may face compounded forms of discrimination, resulting in distinct pathways to CVD. Thus, future work examining biopsychosocial mechanisms in relation to multiple identities could provide important insights into how these overlapping factors contribute to health disparities. In the final section, we discuss these and other ideas for future research aimed at addressing racial inequities in CVD.

## Bridging the Gap: Applying a Multilevel Lens on Discrimination and Racism to Optimize Elucidation of Racial Disparities in CVD Outcomes

The last 30 years have yielded an explosion of research examining racism and discrimination in relation to CVD to better understand the disparate health of African Americans. However, most empirical work and related reviews have centered on interpersonal levels of discrimination and racism in relation to blood pressure. The studies we reviewed herein examined the linkages of racism and discrimination—*as multilevel constructs*—to a fuller complement of critical but understudied clinical and subclinical CVD endpoints. Similar to the literature on discrimination and hypertension, most of the studies in this area have focused on individual-level discrimination or racism as the key exposure. In the aggregate, these studies support links between discrimination and cardiovascular endpoints, with more longitudinal, comprehensive research needed to elucidate the mediators and moderators of these associations. In addition, our review highlights a small but compelling body of work (Appendices C and D) demonstrating adverse impacts of historical, contemporary, and cultural indicators of racism on cardiovascular endpoints, particularly among African Americans. At least four key takeaways may allow us to bridge these gaps in service of the grand objectives of understanding and reducing African Americans’ burden of CVD.

First, simultaneous examination of racism and discrimination as multilevel *and* multidimensional phenomena in relation to CVD risk factors and endpoints is needed."Racism is a system that ensures racial inequality"[35, 161, 162]. Thus, investigating racism as a *system* that functions across all levels of American society is a foundational need of this work. Greater attention to macro-level historical and contemporary sources of structural and institutional racism that result in race-based differences in opportunities, resources, and mobility is essential for *any* work in this area. Public health action attentive to the underlying driving force of structural and in turn, institutional sources of racism while also illuminating its contribution to cultural- and interpersonal-level processes is the best chance we have at reaching health equity. The 2021 AHA Call to Action on Structural Racism and Health Disparities and subsequent 2024 AHA Policy Statement on Structural Racism and Policy Advocacy both address this as a focal and pressing need [9, 17].

Second, careful integration of the role of intergenerational transmission—sensitive to both historical and more contemporary elements—of differential exposure to resources and opportunities for mobility and the related legacy of these processes in communities, groups, and families, is critical to more accurately contextualize the race-related stress burden that African Americans endure [163]. For example, decades of U.S. Census historical tables show that African American children are more likely than White children to come from low-income families, with 79% of African American children experiencing poverty, and 50% for more than 9 years, as compared to 74% and 1%, respectively, of White children; most are raised in single parent homes, have parents with lower levels of education, and are more likely to experience residential instability [164–166]. Undoubtedly, these longstanding early life racial differences are the consequence of enduring racial inequity in housing, education, economic, social, and political structures and policies, while in turn aiding in their contemporary and future perpetuation. Macrolevel structural racism emanating from the built and social environment has served to both compound and generate additional intergenerational losses as well as hinder gains via discriminatory housing practices and neighborhood disinvestment processes, including redlining, serial forced displacement, and targeted closures or placement of amenities and public goods (e.g., schools, pools, and grocery stores). Altogether these factors point to the need to examine structural-level racism to provide context for exploration of *any* other level of racism in relation to CVD health disparities in African Americans.

Third, careful and dedicated examination of a fuller constellation of sociodemographic characteristics—such as sex, age, gender, SES, ableness, ethnicity, place of birth, current region—alongside and in interaction with race and ethnicity is needed. Until the recent emerging interest in intersectionality (i.e., social identities that are inter-dependent, overlapping, and multi-dimensional and likely shaped by historical oppression) in health disparities research, these characteristics were usually treated as covariates and adjusted statistically in analyses, rather than examined as potential effect modifiers that interact with race to shape the types of discriminatory exposures African Americans may face. For instance, an African American woman may achieve high SES as indicated by education, income, and assets, but may face unique exposures to poor treatment due to her gender, sex, and the presence of a visible or invisible disability, thusly, creating an intersectional paradox [58, 59] that would be obscured via a focus solely on race.

Fourth, advancement of cross-disciplinary teams using high-level research methods is needed. Assembly of high-level research teams comprised of individuals with demonstrated commitments to anti-racist and broader diversity, equity and inclusion (DEI) practices—not health equity tourists [167]—across research, mentoring, and clinical settings are needed, such as cardiologists, neuroscientists, radiologists, gerontologists, economists, geographers, anthropologists, and social-behavioral scientists who can develop comprehensive, cutting-edge research that effectively engages the communities of focus. Use of amply sized, cross-region (e.g., rural, suburban, metropolitan) population-based longitudinal cohorts, using sophisticated, repeated measures of racism and discrimination intergenerationally and across an individual’s lifespan is necessary. Given the long window preceding clinical disease, repeated evaluation of subclinical CVD factors alongside assessment of potential multilevel mechanisms is equally critical. In this same vein, methodological approaches that feature a multilevel approach to mediational pathways that explain the linkage of discrimination and racism to CVD or function as “disrupters” to these associations (e.g., strengths-based resources) are necessary to feature more prominently in this body of work as well.

For instance, historically marginalized racial and ethnic groups have long understood the proximal and distal impact of the disadvantage exacted upon their communities—but have yet thrived. In this regard, longstanding sources of resilience and strength for African Americans are likely to include their rich cultural ties and identities and socialization regarding their race, as well as communal supports that offer social capital and sources of emotional, tangible, informational, and instrumental support such as the church, extended familial networks, and community connections via Black Greek lettered organizations (i.e., sororities and fraternities) and other Black-centered organizations (e.g., Jack and Jill, Inc.) [168–171]. Indeed, such sources have helped identify, cull, and pool resources and forge vital connections that promote access and opportunities that transcend generations, enabling sustainability and protection of Black families’ achievements and accomplishments as well as aiding their path to greater upward social mobility [172]. Modeling that employs a multilevel approach that appropriately allows for conceptual breadth and scope reflecting the essence of such multifaceted sources of strength are valuable to begin giving more ongoing attention. In this way, when African Americans seek healthcare, as well as go about their everyday activities (e.g., while shopping, at work, or at school) the translational relevance of understanding what inter- and intra-personal resources they bring to bear upon potentially off-putting challenges linked to structural and institutional inequities they may face, as well as those arising at the interpersonal level can be key to understanding variation in linkages to health disparities we observe within the Black community**.** Finally, examination of such complex relationships not solely between, but also *within,* racial-ethnic groups, primarily African Americans and Native Americans must be a central endeavor of this work [173].

Underlying the above must be an expressed commitment of federal- and state-funding to support this work for as long as it is needed to achieve health equity in CVD outcomes. Such a commitment is critical given widespread mischaracterizations and state-level policies implemented to undermine the mission of DEI-centered work (e.g., see [174, 175]). For instance, NIH’s UNITE initiative—developed in the wake of George Floyd’s murder—is the first time all NIH Institutes and Centers, have collectively focused on structural racism in biomedical science, placing critical resources to undo these associations [176, 177]). Once research is able to empirically address the origins and pathways of the multi-level linkages between racism and CVD, interventions can align with national efforts to rectify historical racist policies in areas like housing, healthcare, education, and employment and, ultimately, promote health equity.

## Overall Conclusion

Racism or race-based discrimination occurs at all levels of society—including structural, institutional, cultural, and interpersonal levels—which operate in conjunction to shorten the life expectancy of African Americans. The available literature reviewed here offers compelling evidence that this linkage can be attributed, at least in part, to enhanced CVD morbidity and mortality. This review strongly suggests robust relations of multilevel racism to both subclinical and clinical manifestations of CVD despite heterogeneous exposure and outcome measures, and identifies links to substantive biopsychosocial pathways to these endpoints. However, much remains to be done, particularly with respect to further operationalization and comprehensive assessment of structural, institutional, and cultural levels of racism alongside measures of interpersonal discriminatory experience, standard assessment of subclinical and clinical CVD, and use of longitudinal designs to elucidate the complex pathways whereby multilevel discrimination and racism promote CVD pathogenesis. Application of intersectional perspectives to both within and between racial group designs will further inform the development of tailored prevention and intervention efforts and most importantly, health, economic, legal, and social policies—responsive to de jure and de facto vestiges of racism—that can eliminate the entrenched cardiovascular health inequity that Black communities continue to endure.

## Key References

- Zestcott CA, Ruiz JM, Tietje KR, Stone J. The relationship between racial prejudice and cardiovascular disease mortality risk at the state and county level. Ann Behav Med 2022;56:959–68. 10.1093/abm/kaab103.
  - Findings from this study suggest that area-level racial prejudice increases CVD mortality risk for likely targets (e.g., Blacks and Native Americans) as well as others (e.g., Whites).
- Deo SV, Motairek I, Nasir K, Mentias A, Elgudin Y, Virani SS, et al. Association between historical neighborhood redlining and cardiovascular outcomes among US veterans with atherosclerotic cardiovascular diseases. JAMA Netw Open 2023;6:e2322727. 10.1001/jamanetworkopen.2023.22727.
  - Findings from this study suggest that living in a historically redlined neighborhood may predict significantly greater risks for major adverse cardiovascular events including myocardial infarction and stroke.

## Supplementary Information

Below is the link to the electronic supplementary material.

Supplementary file1 (DOCX 61 KB)

## Data Availability

No datasets were generated or analysed during the current study.

## Footnotes

1 The terms “African American” and “Black” are used interchangeably throughout this report. This decision is based partly on the use of these terms in the works cited and discussed. We also capitalize the terms “White” and “Black” to reflect that we are discussing a group of people and to be consistent with the capitalization of “African American.”
