# Supplementary material for: Multilevel Racism and Discrimination and Cardiovascular Disease and Related Biopsychosocial Mechanisms: An Integrated Scoping and Literature Review and Future Research Agenda
Source: Curr Cardiol Rep. 2025 Jun 4;27(1):91. doi: 10.1007/s11886-025-02238-3 (PMC12137394; doi:10.1007/s11886-025-02238-3)
Supplement: Supplementary file 1 — Supplementary file1 (DOCX 61 KB) [file 11886_2025_2238_MOESM1_ESM.docx]

**Supplemental Material**

**Multilevel Racism and Discrimination and Cardiovascular Disease and Related Biopsychosocial Mechanisms: An Integrated Scoping and Literature Review and Future Research Agenda**

Danielle L. Beatty Moody, Elizabeth J. Pantesco, Ayla Novruz, Nedelina Tchangalova, Richard C. Sadler, Kellee White, Jason Ashe, Gilbert C. Gee, LaBarron K. Hill, and Shari R. Waldstein

**Table of Contents**

[Appendix A: Search Terms 2](#_Toc168577551)

[Appendix B: Search Strategies 3](#_Toc168577552)

[EBSCO databases 3](#_Toc168577553)

[Public Health (ProQuest) 4](#_Toc168577554)

[Appendix C: List of Included Studies 5](#_Toc168577555)

[Appendix D: List of Excluded Studies with Reasons 8](#_Toc168577556)

[Appendix E: Publication Year of Included Studies 8](#_Toc168577557)

# Appendix A: Search Terms

| Population (P):  *African Americans* | Exposure (E):  *Discrimination* | Outcome (O):  *Cardiovascular disease* |
| --- | --- | --- |
| "African Americans"[Mesh] | Discrimination | "Cardiovascular Diseases/epidemiology"[Mesh] |
| African American | segregation | arterial stiffness |
| African Americans |  | atherosclerosis |
| Black |  | cardiometabolic |
| Blacks |  | cardiovascular |
|  |  | carotid artery |
|  |  | coronary artery obstruction |
|  |  | coronary calcification |
|  |  | coronary obstruction |
|  |  | endothelial dysfunction |
|  |  | heart disease |
|  |  | peripheral arterial disease |
|  |  | stroke |
|  |  | sudden cardiac arrest |
|  |  | sudden cardiac death |
|  |  | transient ischemic attack |
|  |  | white matter hyperintensities |
|  |  | white matter lesion volume |
| A limitation of our scoping review is that we excluded the term "Racism"[MeSH] from the search strategy to maintain a balance between precision and recall, focusing instead on 'discrimination' and 'segregation' within the title and abstract. As a result, relevant studies that specifically address racism may have been inadvertently omitted. However, we supplemented this review with relevant studies identified through hand-searching the references of included studies and through database alerts that included the term 'racism'. | | |

# Appendix B: Search Strategies

## EBSCO databases

**Academic Search Ultimate, APA PsycINFO, CINAHL, MEDLINE**

**Date of search:** January 31, 2024

| Search ID# | Query | Academic Search Ultimate | APA PsycINFO | CINAHL | MEDLINE |
| --- | --- | --- | --- | --- | --- |
| S1 | TI ( "African American" OR "African Americans" OR Black OR Blacks ) OR AB ( "African American" OR "African Americans" OR Black OR Blacks ) OR SU ( "African American" OR "African Americans" OR Black OR Blacks ) | **530,306** | **129,251** | **105,906** | **275,270** |
| S2 | TI ( discrimination OR segregation ) OR AB ( discrimination OR segregation ) OR SU ( discrimination OR segregation ) | **252,586** | **140,064** | **45,469** | **234,455** |
| S3 | TI ( "arterial stiffness" OR atherosclerosis OR cardiometabolic OR cardiovascular OR "coronary artery disease" OR "coronary artery obstruction" OR "coronary calcification" OR “coronary heart disease” OR "coronary obstruction" OR "endothelial dysfunction" OR "heart disease" OR "peripheral arterial disease" OR stroke OR "sudden cardiac arrest" OR "sudden cardiac death" OR "transient ischemic attack" OR "white matter hyperintensities" OR "white matter lesion volume" ) OR AB ( "arterial stiffness" OR atherosclerosis OR cardiometabolic OR cardiovascular OR "coronary artery disease" OR "coronary artery obstruction" OR "coronary calcification" OR “coronary heart disease” OR "coronary obstruction" OR "endothelial dysfunction" OR "heart disease" OR "peripheral arterial disease" OR stroke OR "sudden cardiac arrest" OR "sudden cardiac death" OR "transient ischemic attack" OR "white matter hyperintensities" OR "white matter lesion volume" ) OR SU ( "arterial stiffness" OR atherosclerosis OR cardiometabolic OR cardiovascular OR "coronary artery disease" OR "coronary artery obstruction" OR "coronary calcification" OR “coronary heart disease” OR "coronary obstruction" OR "endothelial dysfunction" OR "heart disease" OR "peripheral arterial disease" OR stroke OR "sudden cardiac arrest" OR "sudden cardiac death" OR "transient ischemic attack" OR "white matter hyperintensities" OR "white matter lesion volume" ) | **641,506** | **94,261** | **406,081** | **1,406,503** |
| S4 | **S1 AND S2 AND S3** | **280** | **188** | **229** | **437** |
| S5 | TI ("blood pressure" OR hypertension) OR AB ("blood pressure" OR hypertension) OR SU ("blood pressure" OR hypertension) | **302,515** | **41,516** | **198,258** | **893,496** |
| S6 | **S4 NOT S5** | **194** | **109** | **147** | **294** |
| S7 | Limiters - Peer Reviewed  Expanders - Apply equivalent subjects  Narrow by Language: - English  Search modes - Find all my search terms | **174** | **81** | **143** | **286** |

**TOTAL: 684 records**

## Public Health (ProQuest)

**Date of search:** January 31, 2024

| Search ID# | Query | Public Health (ProQuest) Results |
| --- | --- | --- |
| S1 | ab("African American" OR "African Americans" OR Black OR Blacks) OR ti("African American" OR "African Americans" OR Black OR Blacks) OR mesh("African American" OR "African Americans" OR Black OR Blacks) | **65,787** |
| S2 | ab(discrimination OR segregation) OR ti(discrimination OR segregation) OR mesh(discrimination OR segregation) | **25,980** |
| S3 | ab("arterial stiffness" OR atherosclerosis OR cardiometabolic OR cardiovascular OR "coronary artery disease” OR "coronary artery obstruction" OR "coronary calcification" OR “coronary heart disease” OR "coronary obstruction" OR "endothelial dysfunction" OR "heart disease" OR "peripheral arterial disease" OR stroke OR "sudden cardiac arrest" OR "sudden cardiac death" OR "transient ischemic attack" OR "white matter hyperintensities" OR "white matter lesion volume") OR ti("arterial stiffness" OR atherosclerosis OR cardiometabolic OR cardiovascular OR "coronary artery disease” OR "coronary artery obstruction" OR "coronary calcification" OR “coronary heart disease” OR "coronary obstruction" OR "endothelial dysfunction" OR "heart disease" OR "peripheral arterial disease" OR stroke OR "sudden cardiac arrest" OR "sudden cardiac death" OR "transient ischemic attack" OR "white matter hyperintensities" OR "white matter lesion volume") OR mesh("arterial stiffness" OR atherosclerosis OR cardiometabolic OR cardiovascular OR "coronary artery disease” OR "coronary artery obstruction" OR "coronary calcification" OR “coronary heart disease” OR "coronary obstruction" OR "endothelial dysfunction" OR "heart disease" OR "peripheral arterial disease" OR stroke OR "sudden cardiac arrest" OR "sudden cardiac death" OR "transient ischemic attack" OR "white matter hyperintensities" OR "white matter lesion volume") | **141,859** |
| S4 | S1 AND S2 AND S3 | **117** |
| S5 | ab("blood pressure" OR hypertension) OR ti("blood pressure" OR hypertension) OR mesh("blood pressure" OR hypertension) | **70,777** |
| S6 | S4 NOT S5 | **85** |
| S7 | **Limiters:** Peer reviewed, English | **80** |

# Appendix C: List of Included Studies

1. Albert, M. A., Cozier, Y., Ridker, P. M., Palmer, J. R., Glynn, R. J., Rose, L., Halevy, N., & Rosenberg, L. (2010). Perceptions of race/ethnic discrimination in relation to mortality among Black women: Results from the Black Women’s Health Study. *Archives of Internal Medicine*, *170*(10), 896–904. <https://doi.org/10.1001/archinternmed.2010.116>
2. Albert, M. A., Ravenell, J., Glynn, R. J., Khera, A., Halevy, N., & de Lemos, J. A. (2008). Cardiovascular risk indicators and perceived race/ethnic discrimination in the Dallas Heart Study. *American Heart Journal*, *156*(6), 1103–1109. <https://doi.org/10.1016/j.ahj.2008.07.027>
3. Ayotte, B. J., Hausmann, L. R. M., Whittle, J., & Kressin, N. R. (2012). The relationship between perceived discrimination and coronary artery obstruction. *American Heart Journal*, *163*(4), 677–683. <https://doi.org/10.1016/j.ahj.2012.01.006>
4. Beatty Moody, D. L., Leibel, D. K., Pantesco, E. J., Wendell, C. R., Waldstein, S. R., Evans, M. K., & Zonderman, A. B. (2020). Interactive relations across dimensions of interpersonal-level discrimination and depressive symptoms to carotid intimal-medial thickening in African Americans. *Psychosomatic Medicine*, *82*(2), 234–246. <https://doi.org/10.1097/PSY.0000000000000765>
5. Beatty Moody, D. L., Taylor, A. D., Leibel, D. K., Al-Najjar, E., Katzel, L. I., Davatzikos, C., Gullapalli, R. P., Seliger, S. L., Kouo, T., Erus, G., Rosenberger, W. F., Evans, M. K., Zonderman, A. B., & Waldstein, S. R. (2019). Lifetime discrimination burden, racial discrimination, and subclinical cerebrovascular disease among African Americans. *Health Psychology*, *38*(1), 63–74. <https://doi.org/10.1037/hea0000638>
6. Bromfield, S. G., Sullivan, S., Saelee, R., Elon, L., Lima, B., Young, A., Uphoff, I., Li, L., Quyyumi, A., Bremner, J. D., Vaccarino, V., & Lewis, T. T. (2020). Race and gender differences in the association between experiences of everyday discrimination and arterial stiffness among patients with coronary heart disease. *Annals of Behavioral Medicine*, *54*(10), 761–770. <https://doi.org/10.1093/abm/kaaa015>
7. Cardarelli, R., Cardarelli, K. M., Fulda, K. G., Espinoza, A., Cage, C., Vishwanatha, J., Young, R., Steele, D. N., & Carroll, J. (2010). Self-reported racial discrimination, response to unfair treatment, and coronary calcification in asymptomatic adults: The North Texas Healthy Heart study. *BMC Public Health*, *10*, 285. <https://doi.org/10.1186/1471-2458-10-285>
8. Carlisle, S. K. (2015). Perceived discrimination and chronic health in adults from nine ethnic subgroups in the USA. *Ethnicity & Health*, *20*(3), 309–326. <https://doi.org/10.1080/13557858.2014.921891>
9. Chae, D. H., Clouston, S., Hatzenbuehler, M. L., Kramer, M. R., Cooper, H. L. F., Wilson, S. M., Stephens-Davidowitz, S. I., Gold, R. S., & Link, B. G. (2015). Association between an internet-based measure of area racism and Black mortality. *PloS One*, *10*(4), e0122963. <https://doi.org/10.1371/journal.pone.0122963>
10. Chae, D. H., Lincoln, K. D., Adler, N. E., & Syme, S. L. (2010). Do experiences of racial discrimination predict cardiovascular disease among African American men? The moderating role of internalized negative racial group attitudes. *Social Science & Medicine*, *71*(6), 1182–1188. <https://doi.org/10.1016/j.socscimed.2010.05.045>
11. Chae, D. H., Nuru-Jeter, A. M., Lincoln, K. D., & Jacob Arriola, K. R. (2012). Racial discrimination, mood disorders, and cardiovascular disease among Black Americans. *Annals of Epidemiology*, *22*(2), 104–111. <https://doi.org/10.1016/j.annepidem.2011.10.009>
12. Cooper, D. C., Mills, P. J., Bardwell, W. A., Ziegler, M. G., & Dimsdale, J. E. (2009). The effects of ethnic discrimination and socioeconomic status on endothelin-1 among Blacks and Whites. *American Journal of Hypertension*, *22*(7), 698–704. <https://doi.org/10.1038/ajh.2009.72>
13. Cuevas, A. G., Ho, T., Rodgers, J., DeNufrio, D., Alley, L., Allen, J., & Williams, D. R. (2021). Developmental timing of initial racial discrimination exposure is associated with cardiovascular health conditions in adulthood. *Ethnicity & Health*, *26*(7), 949–962. <https://doi.org/10.1080/13557858.2019.1613517>
14. Cundiff, J. M., Kamarck, T. W., Muldoon, M. F., Marsland, A. L., & Manuck, S. B. (2023). Expectations of respect and appreciation in daily life and associations with subclinical cardiovascular disease. *Health Psychology*, *42*(1), 53–62. <https://doi.org/10.1037/hea0001255>
15. Deo, S. V., Motairek, I., Nasir, K., Mentias, A., Elgudin, Y., Virani, S. S., Rajagopalan, S., & Al-Kindi, S. (2023). Association between historical neighborhood redlining and cardiovascular outcomes among US veterans with atherosclerotic cardiovascular diseases. *JAMA Network Open*, *6*(7), e2322727. <https://doi.org/10.1001/jamanetworkopen.2023.22727>
16. Dunlay, S. M., Lippmann, S. J., Greiner, M. A., O’Brien, E. C., Chamberlain, A. M., Mentz, R. J., & Sims, M. (2017). Perceived discrimination and cardiovascular outcomes in older African Americans: Insights from the Jackson Heart Study. *Mayo Clinic Proceedings*, *92*(5), 699–709. <https://doi.org/10.1016/j.mayocp.2017.01.024>
17. Everage, N. J., Gjelsvik, A., McGarvey, S. T., Linkletter, C. D., & Loucks, E. B. (2012). Inverse associations between perceived racism and coronary artery calcification. *Annals of Epidemiology*, *22*(3), 183–190. <https://doi.org/10.1016/j.annepidem.2012.01.005>
18. Everson-Rose, S. A., Lutsey, P. L., Roetker, N. S., Lewis, T. T., Kershaw, K. N., Alonso, A., & Diez Roux, A. V. (2015). Perceived discrimination and incident cardiovascular events: The multi-ethnic study of atherosclerosis. *American Journal of Epidemiology*, *182*(3), 225–234. <https://doi.org/10.1093/aje/kwv035>
19. Gavin, A. R., Woo, B., Conway, A., & Takeuchi, D. (2022). The association between racial discrimination, posttraumatic stress disorder, and cardiovascular-related conditions among non-Hispanic Blacks: Results from the National Epidemiologic Survey on Alcohol and Related Conditions-III (NESARC-III). *Journal of Racial and Ethnic Health Disparities*, *9*(1), 193–200. <https://doi.org/10.1007/s40615-020-00943-z>
20. Huang, D., Huang, Y., Adams, N., Nguyen, T. T., & Nguyen, Q. C. (2020). Twitter-characterized sentiment towards racial/ethnic minorities and Cardiovascular Disease (CVD) outcomes. *Journal of Racial and Ethnic Health Disparities*, *7*(5), 888–900. <https://doi.org/10.1007/s40615-020-00712-y>
21. Kramer, M. R., Black, N. C., Matthews, S. A., & James, S. A. (2017). The legacy of slavery and contemporary declines in heart disease mortality in the U.S. South. *SSM - Population Health*, *3*, 609–617. <https://doi.org/10.1016/j.ssmph.2017.07.004>
22. Lawrence, W. R., Jones, G. S., Johnson, J. A., Ferrell, K. P., Johnson, J. N., Shiels, M. S., Diez Roux, A. V., & Forde, A. T. (2023). Discrimination experiences and all-cause and cardiovascular mortality: Multi-ethnic study of atherosclerosis. *Circulation. Cardiovascular Quality and Outcomes*, *16*(4), e009697. <https://doi.org/10.1161/CIRCOUTCOMES.122.009697>
23. Leitner, J. B., Hehman, E., Ayduk, O., & Mendoza-Denton, R. (2016). Racial bias is associated with ingroup death rate for Blacks and Whites: Insights from Project Implicit. *Social Science & Medicine*, S0277-9536(16)30566-4. <https://doi.org/10.1016/j.socscimed.2016.10.007>
24. Lewis, T. T., Everson-Rose, S. A., Powell, L. H., Matthews, K. A., Brown, C., Karavolos, K., Sutton-Tyrrell, K., Jacobs, E., & Wesley, D. (2006). Chronic exposure to everyday discrimination and coronary artery calcification in African-American women: The SWAN Heart Study. *Psychosomatic Medicine*, *68*(3), 362–368. <https://doi.org/10.1097/01.psy.0000221360.94700.16>
25. Lewis, T. T., Lampert, R., Charles, D., & Katz, S. (2019). Expectations of racism and carotid intima-media thickness in African American women. *Psychosomatic Medicine*, *81*(8), 759–768. <https://doi.org/10.1097/PSY.0000000000000684>
26. Lukachko, A., Hatzenbuehler, M. L., & Keyes, K. M. (2014). Structural racism and myocardial infarction in the United States. *Social Science & Medicine*, *103*, 42–50. <https://doi.org/10.1016/j.socscimed.2013.07.021>
27. Mouzon, D. M., Taylor, R. J., Woodward, A., & Chatters, L. M. (2017). Everyday racial discrimination, everyday non-racial discrimination, and physical health among African Americans. *Journal of Ethnic & Cultural Diversity in Social Work*, *26*(1–2), 68–80. <https://doi.org/10.1080/15313204.2016.1187103>
28. Nardone, A., Casey, J. A., Morello-Frosch, R., Mujahid, M., Balmes, J. R., & Thakur, N. (2020). Associations between historical residential redlining and current age-adjusted rates of emergency department visits due to asthma across eight cities in California: An ecological study. *The Lancet. Planetary Health*, *4*(1), e24–e31. <https://doi.org/10.1016/S2542-5196(19)30241-4>
29. Okhomina, V. I., Glover, L., Taylor, H., & Sims, M. (2018). Dimensions of and responses to perceived discrimination and subclinical disease among African-Americans in the Jackson Heart Study. *Journal of Racial and Ethnic Health Disparities*, *5*(5), 1084–1092. <https://doi.org/10.1007/s40615-017-0457-7>
30. Peterson, L. M., Matthews, K. A., Derby, C. A., Bromberger, J. T., & Thurston, R. C. (2016). The relationship between cumulative unfair treatment and intima media thickness and adventitial diameter: The moderating role of race in the study of women’s health across the nation. *Health Psychology*, *35*(4), 313–321. <https://doi.org/10.1037/hea0000288>
31. Sheehy, S., Aparicio, H. J., Palmer, J. R., Cozier, Y., Lioutas, V.-A., Shulman, J. G., & Rosenberg, L. (2023). Perceived interpersonal racism and incident stroke among US Black women. *JAMA Network Open*, *6*(11), e2343203. <https://doi.org/10.1001/jamanetworkopen.2023.43203>
32. Splan, E. D., Magerman, A. B., & Forbes, C. E. (2021). Associations of regional racial attitudes with chronic illness in the United States. *Social Science & Medicine*, *281*, 114077. <https://doi.org/10.1016/j.socscimed.2021.114077>
33. Troxel, W. M., Matthews, K. A., Bromberger, J. T., & Sutton-Tyrrell, K. (2003). Chronic stress burden, discrimination, and subclinical carotid artery disease in African American and Caucasian women. *Health Psychology*, *22*(3), 300–309. <https://doi.org/10.1037/0278-6133.22.3.300>
34. Udo, T., & Grilo, C. M. (2017). Cardiovascular disease and perceived weight, racial, and gender discrimination in U.S. adults. *Journal of Psychosomatic Research*, *100*, 83–88. <https://doi.org/10.1016/j.jpsychores.2017.07.007>
35. Whaley, A. L. (2022). Ethnicity, nativity, and the effects of stereotypes on cardiovascular health among people of African ancestry in the United States: Internal versus external sources of racism. *Ethnicity & Health*, *27*(5), 1010–1030. <https://doi.org/10.1080/13557858.2020.1847257>
36. Zahodne, L. B., Sharifian, N., Kraal, A. Z., Morris, E. P., Sol, K., Zaheed, A. B., Meister, L., Mayeux, R., Schupf, N., Manly, J. J., & Brickman, A. M. (2023). Longitudinal associations between racial discrimination and hippocampal and white matter hyperintensity volumes among older Black adults. *Social Science & Medicine*, *316*, 114789. <https://doi.org/10.1016/j.socscimed.2022.114789>
37. Zestcott, C. A., Ruiz, J. M., Tietje, K. R., & Stone, J. (2022). The relationship between racial prejudice and cardiovascular disease mortality risk at the state and county level. *Annals of Behavioral Medicine*, *56*(9), 959–968. <https://doi.org/10.1093/abm/kaab103>

# Appendix D: List of Excluded Studies with Reasons

*Reason for Exclusion: Wrong Population*

Fluck, D., Fry, C. H., Gulli, G., Affley, B., Robin, J., Kakar, P., Sharma, P., & Han, T. S. (2023). Adverse stroke outcomes amongst UK ethnic minorities: A multi-centre registry-based cohort study of acute stroke. *Neurological Sciences : Official Journal of the Italian Neurological Society and of the Italian Society of Clinical Neurophysiology*, *44*(6), 2071–2080. cmedm. <https://doi.org/10.1007/s10072-023-06640-z>

Maschino, L., Cook, S., & Lucas, T. (2024). Personal justice beliefs, everyday discrimination, and carotid intima media thickness in sexual minority men. *Health Psychology*, *43*(1), 1–6. psyh. <https://doi.org/10.1037/hea0001329>

Yu, S., Tavarez-Mora, F., Milam, A. J., Misra, L., Aljure, O., Glas, K., & Shillcutt, S. (2023). Matters of the Heart: Examining Motivating Factors and Unconscious Bias in the Adult Cardiothoracic Anesthesiology Fellowship. *Journal of Cardiothoracic and Vascular Anesthesia*, *37*(7), 1160–1168. cmedm. <https://doi.org/10.1053/j.jvca.2023.02.044>

*Reason for Exclusion: Wrong Exposure*

Barforoshi, S., Manubolu, V. S., Wang, R., McClelland, R. L., & Budoff, M. J. (2024). Incremental Value of ABI and CAC Beyond Traditional Risk Markers in Long-term Prediction of Cardiovascular Disease Incidence in Participants with Diabetes: Multi-Ethnic Study of Atherosclerosis. *The American Heart Journal*, *267*, 117–118. <https://doi.org/10.1016/j.ahj.2023.08.017>

Bikomeye, J. C., Awoyinka, I., Kwarteng, J. L., Beyer, A. M., Rine, S., & Beyer, K. M. M. (2024). Disparities in Cardiovascular Disease-Related Outcomes Among Cancer Survivors in the United States: A Systematic Review of the Literature. *Heart, Lung & Circulation*. cmedm. <https://doi.org/10.1016/j.hlc.2023.11.003>

Deets, A., Joshi, P. H., Chandra, A., Singh, K., Khera, A., Virani, S. S., Ballantyne, C. M., Otvos, J. D., Dullaart, R. P. F., Gruppen, E. G., Connelly, M. A., Ayers, C., Navar, A. M., Pandey, A., Wilkins, J. T., & Rohatgi, A. (2023). Novel Size-Based High-Density Lipoprotein Subspecies and Incident Vascular Events. *Journal of the American Heart Association*, *12*(21), e031160. cmedm. <https://doi.org/10.1161/JAHA.123.031160>

Dixon, D. D., & Wilkins, C. H. (2023). Strategies to Cultivate Diversity and Achieve Equity in Cardiovascular Clinical Trials. *Circulation*, *148*(3), 204–206. asn.

Doan, S. N., Davis, A. S., Lazarus, M., Poudel, A., Tran, P., Clark, N., & Fuller-Rowell, T. E. (2023). Belonging Exacerbates the Relations Between Racial Climate Stress and Physiological Dysregulation. *Journal of Racial and Ethnic Health Disparities*. cmedm. <https://doi.org/10.1007/s40615-023-01740-0>

Ghosh, A. K., Venkatraman, S., Nanna, M. G., Safford, M. M., Colantonio, L. D., Brown, T. M., Pinheiro, L. C., Peterson, E. D., Navar, A. M., Sterling, M. R., Soroka, O., Nahid, M., Banerjee, S., & Goyal, P. (2024). Risk Prediction for Atherosclerotic Cardiovascular Disease With and Without Race Stratification. *JAMA Cardiology*, *9*(1), 55–62. cmedm. <https://doi.org/10.1001/jamacardio.2023.4520>

Rattanawong, P., Mattanapojanat, N., Mead-Harvey, C., Van Der Walt, C., Kewcharoen, J., Kanitsoraphan, C., Vutthikraivit, W., Prasitlumkum, N., Putthapiban, P., Chintanavilas, K., Sahasthas, D., Ngarmukos, T., Thakkinstian, A., Sorajja, D., Makarawate, P., & Shen, W.-K. (2023). Predicting arrhythmic event score in Brugada syndrome: Worldwide pooled analysis with internal and external validation. *Heart Rhythm*, *20*(10), 1358–1367. cmedm. <https://doi.org/10.1016/j.hrthm.2023.06.013>

Reddy, K. P., Eberly, L. A., Julien, H. M., Giri, J., Fanaroff, A. C., Groeneveld, P. W., Khatana, S. A. M., & Nathan, A. S. (2023). Association between racial residential segregation and Black-White disparities in cardiovascular disease mortality. *American Heart Journal*, *264*, 143–152. <https://doi.org/10.1016/j.ahj.2023.06.010>

Reddy, S. M., Wiecha, N., Nguyen, C. T., & Barch, D. H. (2023). The role of adverse pregnancy outcomes in conventional cardiovascular risk prediction. *Maternal and Child Health Journal*, *27*(10), 1774–1786. psyh. <https://doi.org/10.1007/s10995-023-03725-1>

Schwartz, B. N., Evans, F. J., Burns, K. M., & Kaltman, J. R. (2023). Social inequities impact infant mortality due to congenital heart disease. *Public Health (Elsevier)*, *224*, 66–73. asn.

*Reason for Exclusion: Wrong Outcome*

Bancks, M. P., Byrd, G. S., Caban-Holt, A., Fitzpatrick, A. L., Forrester, S. N., Hayden, K. M., Heckbert, S. R., Kershaw, K. N., Rapp, S. R., Sachs, B. C., & Hughes, T. M. (2023). Self-reported experiences of discrimination and incident dementia. *Alzheimer’s & Dementia : The Journal of the Alzheimer’s Association*, *19*(7), 3119–3128. cmedm. <https://doi.org/10.1002/alz.12947>

Besser, L. M., Meyer, O. L., Jones, M. R., Tran, D., Booker, M., Mitsova, D., Peterson, R., Galvin, J. E., Bateman, J. R., Hayden, K. M., & Hughes, T. M. (2023). Neighborhood segregation and cognitive change: Multi-Ethnic Study of Atherosclerosis. *Alzheimer’s & Dementia : The Journal of the Alzheimer’s Association*, *19*(4), 1143–1151. cmedm. <https://doi.org/10.1002/alz.12705>

Daniel, D., Maillie, L., & Dhamoon, M. (2023). Provider care segregation and hospital-region racial disparities for carotid interventions in the USA. *Journal of Neurointerventional Surgery*. cmedm. <https://doi.org/10.1136/jnis-2023-020656>

Daniel, D., Maillie, L., & Dhamoon, M. S. (2024). Provider Care Segregation and Hospital-Region Racial Disparities in the United States for Acute Ischemic Stroke and Endovascular Therapy Outcomes. *Journal of the American Heart Association*, e029255. cmedm. <https://doi.org/10.1161/JAHA.122.029255>

Dark, H. E., Huang, A., Cordon, J., Deal, J. A., Palta, P., Windham, B. G., Barnes, L. L., Kucharska‐Newton, A., Mosley, T., Gottesman, R. F., Sims, M., Griswold, M., Arce Rentería, M., Manly, J. J., & Walker, K. A. (2023). The association of perceived discrimination with dementia risk in Black older adults. *Alzheimer’s & Dementia: The Journal of the Alzheimer’s Association*, *19*(10), 4346–4356. psyh. <https://doi.org/10.1002/alz.13135>

Davidson, J. C., Kent, B. V., Cozier, Y. C., Kanaya, A. M., Warner, E. T., Eliassen, A. H., Williams, D. R., & Shields, A. E. (2023). “Does Religious Service Attendance Modify the Relationship between Everyday Discrimination and Risk of Obesity? Results from the Study on Stress, Spirituality and Health”. *Journal of Racial and Ethnic Health Disparities*. cmedm. <https://doi.org/10.1007/s40615-023-01765-5>

Hicken, M. T., Dou, J., Kershaw, K. N., Liu, Y., Hajat, A., & Bakulski, K. M. (2023). Racial and Ethnic Residential Segregation and Monocyte DNA Methylation Age Acceleration. *JAMA Network Open*, *6*(11), e2344722–e2344722. <https://doi.org/10.1001/jamanetworkopen.2023.44722>

Joshi, A., Wilson, L. E., Pinheiro, L. C., Judd, S., & Akinyemiju, T. (2023). Association of racial residential segregation with all-cause and cancer-specific mortality in the reasons for geographic and racial differences in stroke (REGARDS) cohort study. *SSM - Population Health*, *22*, 101374. cmedm. <https://doi.org/10.1016/j.ssmph.2023.101374>

Krobath, D. M., Cuevas, A. G., Allen, J. D., Chung, M., Economos, C. D., & Mistry, J. (2023). The Influence of Contested Racial Identity and Perceived Everyday Discrimination Exposure on Body Mass Index in US Adults. *Journal of Racial and Ethnic Health Disparities*. cmedm. <https://doi.org/10.1007/s40615-023-01774-4>

Lin, S. C., Hammond, G., Esposito, M., Majewski, C., Foraker, R. E., & Joynt Maddox, K. E. (2023). Segregated Patterns of Hospital Care Delivery and Health Outcomes. *JAMA Health Forum*, *4*(11), e234172. cmedm.

Morris, M. C., Moradi, H., Aslani, M., Sims, M., Schlundt, D., Kouros, C. D., Goodin, B., Lim, C., & Kinney, K. (2023). Predicting incident cardiovascular disease among African-American adults: A deep learning approach to evaluate social determinants of health in the Jackson heart study. *PLoS ONE*, *18*(11), 1–18. asn.

Pichardo, M. S., Ferrucci, L. M., Molina, Y., Esserman, D. A., & Irwin, M. L. (2023). Structural Racism, Lifestyle Behaviors, and Obesity-related Cancers among Black and Hispanic/Latino Adults in the United States: A Narrative Review. *Cancer Epidemiology, Biomarkers & Prevention : A Publication of the American Association for Cancer Research, Cosponsored by the American Society of Preventive Oncology*, *32*(11), 1498–1507. cmedm. <https://doi.org/10.1158/1055-9965.EPI-22-1147>

Wolfgang, M., Beskow, L., Hooker, G., Roberson, M., & Anderson, K. (2023). Stigma manifestations in cardiomyopathy care impact outcomes for black patients: A qualitative study. *BMC Cardiovascular Disorders*, *23*(1), 553. cmedm. <https://doi.org/10.1186/s12872-023-03556-6>

*Reason for Exclusion: Wrong Exposure & Outcome*

Andel, R., Veal, B. M., Howard, V. J., MacDonald, L. A., Judd, S. E., & Crowe, M. (2023). Retirement and cognitive aging in a racially diverse sample of older Americans. *Journal of the American Geriatrics Society*, *71*(9), 2769–2778.

Bishop-Royse, J., Saiyed, N. S., Schober, D. J., Laflamme, E., Lange-Maia, B. S., Ferrera, M., & Benjamins, M. R. (2023). Cause-Specific Mortality and Racial Differentials in Life Expectancy, Chicago 2018-2019. *Journal of Racial and Ethnic Health Disparities*. cmedm. <https://doi.org/10.1007/s40615-023-01566-w>

Byrne, M. E., Burke, N. L., Neyland, M. K. H., Bloomer, B. F., Hayes, H. E., Loch, L. K., Te-Vazquez, J., Nwosu, E. E., Lazareva, J., Moursi, N. A., Schvey, N. A., Shomaker, L. B., Brady, S. M., Sbrocco, T., & Tanofsky-Kraff, M. (2023). Negative affect and loss-of-control eating in relation to adiposity among non-Hispanic youth identifying as black or white. *Eating Behaviors*, *49*, N.PAG-N.PAG. asn.

Deo, S. V., Althouse, A., Al-Kindi, S., McAllister, D. A., Orkaby, A., Elgudin, Y. E., Fremes, S., Chu, D., Visseren, F. L. J., Pell, J. P., & Sattar, N. (2023). Validating the SMART2 Score in a Racially Diverse High-Risk Nationwide Cohort of Patients Receiving Coronary Artery Bypass Grafting. *Journal of the American Heart Association*, *12*(21), e030757. cmedm. <https://doi.org/10.1161/JAHA.123.030757>

Lu, X., Gao, R., & Liao, S. (2023). Creatinine-based European Kidney Function Consortium equation is superior to the Chronic Kidney Disease Epidemiology Collaboration equation for mortality risk stratification in general non-Black population. *European Journal of Preventive Cardiology*, *30*(15), 1654–1664. cmedm. <https://doi.org/10.1093/eurjpc/zwad148>

Pearl, R. L., Wadden, T. A., Bach, C., LaFata, E. M., Gautam, S., Leonard, S., Berkowitz, R. I., Latner, J. D., & Jakicic, J. M. (2023). Long-Term Effects of an Internalized Weight Stigma Intervention: A Randomized Controlled Trial. *Journal of Consulting & Clinical Psychology*, *91*(7), 398–410.

Sherman-Hahn, S., Izkhakov, E., Perlman, S., & Ziv-Baran, T. (2023). A new metabolic syndrome prediction model for self-evaluation as a primary screening tool in an apparently MetS-free population. *Preventive Medicine*, *175*, N.PAG-N.PAG. asn.

Metabolic syndrome (MetS) is a growing global public health concern associated with

Shrestha, S., Zhu, X., Sullivan, K. J., Blackshear, C., Deal, J. A., Sharrett, A. R., Kamath, V., Schneider, A. L. C., Jack, C. R., Huang, J., Palta, P., Reid, R. I., Knopman, D. S., Gottesman, R. F., Chen, H., Windham, B. G., Griswold, M. E., & Mosley, T. H. Jr. (2023). Association of olfaction and microstructural integrity of brain tissue in community-dwelling adults: Atherosclerosis risk in communities neurocognitive study. *Neurology*, *101*(13), e1328–e1340. psyh. <https://doi.org/10.1212/WNL.0000000000207636>

Stafford, I. A., Viertel, V. G., Wilken, L. A., Olmsted, K. E., Porter, M. R., Armstrong, J. M., Go, G., & Roberts, A. W. (2023). Health Care Disparities and the Emergency Management of Postpartum Patients with Cardiovascular Complaints. *American Journal of Perinatology*. cmedm. <https://doi.org/10.1055/a-2099-8662>

Sun, F., Yao, J., Du, S., Qian, F., Appleton, A. A., Tao, C., Xu, H., Liu, L., Dai, Q., Joyce, B. T., Nannini, D. R., Hou, L., & Zhang, K. (2023). Social Determinants, Cardiovascular Disease, and Health Care Cost: A Nationwide Study in the United States Using Machine Learning. *Journal of the American Heart Association*, *12*(5), e027919. cmedm. <https://doi.org/10.1161/JAHA.122.027919>

Tian, J., Yan, J., Han, G., Du, Y., Hu, X., He, Z., Han, Q., & Zhang, Y. (2023). Machine learning prognosis model based on patient-reported outcomes for chronic heart failure patients after discharge. *Health & Quality of Life Outcomes*, *21*(1), 1–11. asn.

Tong, L., Sun, Y., Zhu, Y., Luo, H., Wan, W., & Wu, Y. (2023). Prognostic estimation for acute ischemic stroke patients undergoing mechanical thrombectomy within an extended therapeutic window using an interpretable machine learning model. *Frontiers in Neuroinformatics*, *17*, 1273827. cmedm. <https://doi.org/10.3389/fninf.2023.1273827>

*Reason for Exclusion: Qualitative*

Knauer, C. (2023). “With a Stroke of a Pen”: Executive Order 9981 in American Memory. *History & Memory*, *35*(2), 41–78. asn.

# Appendix E: Publication Year of Included Studies
